# Bacteriome and mycobiome profiling of liquid feed for finisher pigs on commercial pig farms

**DOI:** 10.1038/s41598-025-05928-8

**Published:** 2025-07-09

**Authors:** J. T. Cullen, P. G. Lawlor, P. Cormican, F. Crispie, H. Slattery, G. E. Gardiner

**Affiliations:** 1 Eco-Innovation Research Centre, Department of Science, South East Technological University, Cork Road Campus, County Waterford, X91 K0EK Ireland; 2 Teagasc Pig Development Department, Animal and Grassland Research and Innovation Centre, Moorepark, Fermoy, County Cork, P61 C996 Ireland; 3 Animal and Bioscience Research Department, Animal and Grassland Research and Innovation Centre, Teagasc, Grange, Dunsany, County Meath, C15 PW93 Ireland; 4 Teagasc Food Research Centre, Moorepark, Fermoy, County Cork, P61 C996 Ireland

**Keywords:** Applied microbiology, Microbiome

## Abstract

The aim was to assess the quality of liquid feed for grow-finisher pigs across commercial pig farms by profiling the bacteriome and mycobiome of samples and determining biogenic amine concentrations. Amplicon sequencing of liquid feed samples revealed that bacterial and fungal community structures were influenced by the farm of origin and sampling location (mixing tank/trough) on a given farm. Decreases in alpha-diversity of liquid feed between the mixing tank and the troughs corresponded with increased relative abundances of bacteria, particularly *Lactobacillus*, *Weissella* and *Leuconostoc,* as well as yeasts, including *Kazachstania* and *Dipodascus*, indicative of spontaneous fermentation. The concentration of biogenic amines, resulting from amino acid loss from the feed, which likely plays a role in poorer feed efficiency, also increased between the mixing tank and the troughs. The highest biogenic amine concentrations in the feed were found on the farm that had the highest lactic acid bacteria (LAB) and yeast counts. Both *Lactobacillus* and *Kazachstania* were correlated with biogenic amine concentrations in liquid feed, highlighting the unexplored role that LAB and yeast may play in amino acid decarboxylation and biogenic amine formation in liquid feed. Factors including the use of liquid co-products in diets also impacted the liquid feed microbiome.

**Supplementary Information:**

The online version contains supplementary material available at 10.1038/s41598-025-05928-8.

## Introduction

Considering that ~ 70% of pigs in Ireland are fed liquid feed as opposed to dry feed^1^, it is important that both the microbial and nutritional quality of liquid feed are optimised to improve growth and feed efficiency (FE). The need to optimise liquid feed quality stems primarily from the observation of unintentional ‘spontaneous’ fermentation occurring in fresh (i.e. not deliberately fermented) liquid feed, which can have negative impacts. For example, amino acid and gross energy losses from the diet have been attributed to spontaneous fermentation in fresh liquid feed^2,3^. This may, at least in part, explain the poorer feed conversion efficiency (FCE) observed in liquid-fed pigs, especially with ad libitum liquid feeding where residual feed remains in the trough for relatively long periods of time^4,5^.

Additionally, microbial decarboxylation of amino acids can lead to the production of biogenic amines, which may impact feed palatability and potentially pig health^2,6,7^. A survey of French finisher farms feeding liquid feed found that concentrations of biogenic amines in liquid feed may be of concern, particularly cadaverine, which is a product of lysine decarboxylation^8,9^. Another potential negative impact of feed fermentation is decreased lipid absorption and energy harvest in pigs due to overgrowth of bile salt hydrolase-producing bacteria (e.g. strains of *Lactobacillus* and *Clostridium*) in the feed and hence potentially in the intestine^10,11^. Although the microbial communities of liquid feed have been relatively well-documented, studies to date have almost exclusively been culture-based, investigating only a limited number of microbial groups. In particular, information regarding the fungal communities in liquid feed is relatively scarce, especially considering the potentially negative impact of yeasts and moulds. Therefore, the objectives of this study were: (1) to profile the bacteriome and (for the first time) the mycobiome of liquid feed from the finisher section of commercial pig farms, using high-throughput 16S rRNA and ITS2 amplicon sequencing, respectively; (2) to determine biogenic amine concentrations as an indication of the level of spontaneous fermentation occurring and to assess feed safety; and (3) to correlate differentially abundant microbial taxa with microbial metabolite concentrations i.e. biogenic amines, volatile fatty acids (VFAs), ethanol and lactate, in the same liquid feed samples.

## Materials and methods

### Sample collection

Sampling of liquid feed was performed by O’Meara et al.^3^ in the finisher section of eight commercial pig farms in Ireland. Information on liquid feeding system practices and the diets fed on each farm are available in Supplementary Tables S1 and S2. A detailed description of the sampling procedures is provided in the O’Meara et al.^3^ publication. Briefly, liquid feed was sampled from the mixing tanks and feed troughs on seven pig farms (Farms A to G) on one sampling occasion, while one farm (Farm H, a research farm) was sampled on seven different occasions. Seven liquid feed samples were collected from three sampling locations on each farm. Firstly, freshly prepared feed was collected from the mixing tank after agitation (*n* = 1). Samples of fresh liquid feed were then collected immediately after delivery to different troughs (*n* = 3), and lastly, samples of residual liquid feed, remaining in the different troughs, were taken just before the next feed-out (*n* = 3). On Farm D, the troughs did not contain residual feed and therefore, only mixing tank and fresh trough samples were taken on this farm ^3^. In addition, on four of the seven sampling occasions on Farm H, only two fresh and residual trough samples could be collected, in addition to the one mixing tank sample. Liquid feed samples for bacteriome and mycobiome analysis were transferred aseptically into 1.5 mL sterile Eppendorf tubes on-farm. Samples were transported to the laboratory on dry ice, snap-frozen in liquid nitrogen upon arrival and stored at − 80 °C until DNA extraction. Liquid feed sub-samples (~ 20 g) from the three sampling locations of 6 of the farms (Farms B, C, E, F, G and H) were also collected by O’Meara et al.^3^, as described for lactate, ethanol and VFA analysis sampling in their study, and stored at − 20 °C for biogenic amine analysis.

### Bacteriome and mycobiome analysis of liquid feed samples

#### DNA extraction

DNA extraction from liquid feed samples was performed using the QIAamp® Fast DNA Stool Mini kit, following the ‘Isolation of DNA from Stool for Pathogen Detection’ protocol. Previously described modifications to this procedure were followed, which included a 20 min bead-beating step for simultaneous extraction of bacterial and fungal DNA as optimised by Cullen et al.^12^. The Qubit® 3.0 Fluorometer was used for DNA quantification with the Qubit® dsDNA HS Assay Kit (Bio-Sciences, Dublin, Ireland).

#### Library preparation and amplicon sequencing

Bacterial communities were profiled via amplicon sequencing of the V3-V4 hypervariable region of the 16S rRNA gene on the Illumina MiSeq platform, according to the Illumina 16S Metagenomic Sequencing Library Preparation Guide, with some modifications, as described by Cullen et al.^12^. The V3-V4 region was amplified using primers S-D-Bact-0341-b-S-17/S-D-Bact-0785-a-A-21 from Klindworth et al.^13^. Fungal profiling was performed by amplifying the nuclear ribosomal ITS2 region with the ITS3 and ITS4 primer set from White et al.^14^. Each PCR reaction contained 50 ng of DNA template extracted from feed samples. The reaction volume, components and PCR conditions were the same as those described by Cullen et al.^12^. The 16S and ITS2 PCR products were quantified using the Qubit® 3.0 Fluorometer as described above, quality checked and purified as described previously^12^. Final libraries were quantified by qPCR, diluted, denatured and sequenced using a 2 × 300 cycle V3 kit for the 16S library and a 2 × 250 cycle V2 kit for the ITS2 library in the Teagasc sequencing facility as described by Fouhy et al.^15^ in accordance with standard Illumina sequencing protocols.

### Bioinformatics and statistical analysis

Raw FASTQ files for this study have been deposited in the European Nucleotide Archive (ENA) at EMBL-EBI under project accession number PRJEB72728 (https://www.ebi.ac.uk/ena/browser/view/PRJEB72728). Demultiplexed paired-end 16S and ITS2 rDNA sequences were imported (in Casava 1.8 demultiplexed paired-end format) into QIIME2 v.2020.8.0^16^. Sequence quality assessment and initial pre-processing including primer trimming, filtering, dereplication, chimera removal, and merging of paired-end reads were performed in QIIME2 as described previously^12^, except that Read 1 and Read 2 of the 16S rDNA sequences were truncated at 255 and 199 bp, respectively, and Read 1 and Read 2 of the ITS2 sequences were truncated at 219 and 172 bp, respectively. Taxonomic assignment for bacterial and fungal ASVs was performed using classifiers previously trained on sequences from the SILVA (version 138_99)^17^ and UNITE (version 8_99)^18^ databases, respectively^12^.

QIIME artefacts (taxonomy, ASV table, metadata and phylogenetic tree) were imported into R (version 4.2.1) as a phyloseq^19^ object with the qza_to_phyloseq function in the qiime2r package^20^. Contaminant bacterial and fungal ASVs, identified using the ‘prevalence’ method in the decontam package^21^, were removed prior to further analysis. Further pre-processing included removal of ASVs that were not assigned to the kingdoms *Bacteria* and *Fungi*, for each respective dataset, and removal of ASVs that phylum-level taxonomy was not assigned to. Finally, the filter_taxa function in phyloseq was used to remove ASVs that were not observed more than 3 times in at least 20% of the samples, for each respective dataset (i.e. 16S and ITS2 datasets).

Alpha-diversity (observed ASVs, Pielou’s evenness and Shannon diversity) and beta-diversity, based on unrarefied filtered sequences, were calculated using the phyloseq package. Differences in alpha-diversity metrics were analysed using a linear mixed-effects model using the lmer function in the lme4 package^22^, with farm as a random effect. Statistical significance between sampling locations was tested using the ANOVA function in the car package, followed by pairwise comparisons using Tukey’s HSD test with the emmeans package^23^. Alpha-diversity was plotted using the ggpubr package^24^. Beta-diversity was measured using non-metric multidimensional scaling (NMDS) of Bray–Curtis dissimilarity distances, and was plotted using the ggplot2 package^25^. Permutational multivariate analysis of variance (PERMANOVA) with 10,000 permutations was performed to test for differences between samples using the adonis2 function in the vegan package^26^.

The ancombc2 function in the analysis of compositions of microbiomes with bias correction (ANCOMBC) package^27^ was used to identify differentially abundant bacterial and fungal genera between sampling locations in liquid feed. Differential abundance was expressed as log-fold changes, which represent the difference in bias-corrected abundances between groups. Default ancombc2 settings were used unless otherwise specified. In order to avoid spurious results, ‘prv_cut’ was set to 0.65 and 0.60 for the bacterial and fungal datasets, respectively. Therefore, genera that were present in less than 65 and 60% of samples for the bacterial and fungal datasets, respectively, were removed prior to analysis. Pairwise comparisons between sampling locations were tested using the pairwise directional test, with farm included in the model as a random effect using the ‘rand_formula’ option. Genera with an adjusted *p*-value of ≤ 0.05 were considered differentially abundant and log-fold changes of pairwise comparisons were plotted using ggplot2^25^.

Pearson correlations between differentially abundant bacterial and fungal genera were performed against biogenic amine, lactate, ethanol and VFA concentrations in the same liquid feed samples. Existing lactate, ethanol and VFA data, generated by O’Meara et al.^3^ for the same samples, were used for correlation analysis, as well as biogenic amine data generated in the current study as outlined below. Correlations were performed in R using the microeco package^28^ after converting the phyloseq objects to microtable objects using the file2meco package^29^. The trans_diff class was used to perform differential abundance testing at the genus level using the linear discriminant analysis (LDA) effect size (LEfSe) method with default settings, except that ASVs below 0.5% relative abundance were removed to avoid spurious results. A trans_env object containing the biogenic amine, lactate, ethanol and VFA data was generated and correlated with the genus level differential abundance data using the cal_cor function, using false discovery rate multiple testing correction.

### Extraction, derivatisation and HPLC analysis of biogenic amines

Extraction of biogenic amines from the liquid feed samples taken from the mixing tank and troughs was performed based on the method of Yoon et al.^30^ with modifications. Liquid feed samples, which had been stored at − 20 °C, were defrosted at room temperature and ~ 20 g was homogenised for 3 min using a DI 25 Basic homogeniser (IKA, Königswinter, Germany) at 13,500 rpm. Briefly, 5 g of liquid feed homogenate was weighed and 3 mL of 2% trichloroacetic acid (TCA) (Sigma-Aldrich, Wicklow, Ireland) was added, followed by homogenisation for 30 s at 13,500 rpm. Samples were reacted at 4 °C for 2 h with shaking, followed by centrifugation (4000 rpm) at 4 °C for 20 min. The supernatant was added to a 10 mL volumetric flask. The residue was then re-extracted with another 3 mL of 2% TCA, following the same procedure, except that the second extract was reacted at 4 °C for 1 h. Both supernatants were then pooled and diluted to a final volume of 10 mL with 2% TCA and stored at − 20 °C until derivatisation and HPLC analysis.

Benzoyl chloride derivatisation of biogenic amines and HPLC analysis were performed as described by O’Sullivan et al.^31^, with minor modifications, as follows. A Waters® Alliance 2695 HPLC System equipped with a 2487 Dual Absorbance Detector (Waters Chromatography, Dublin, Ireland) was employed with an injection volume of 10 µL used for samples and standards. Biogenic amines were quantified using calibration curves generated from 5 standard solution mixtures containing histamine (2.5–50 μg/mL), putrescine (1.25–5 μg/mL), cadaverine (1.25–25 μg/mL), tyramine (2.5–50 μg/mL) and tryptamine (2.5–50 μg/mL) (Sigma-Aldrich) (Supplementary Table S3).

## Results

### Diversity of bacteria and fungi in liquid feed

**Fig. 1:**
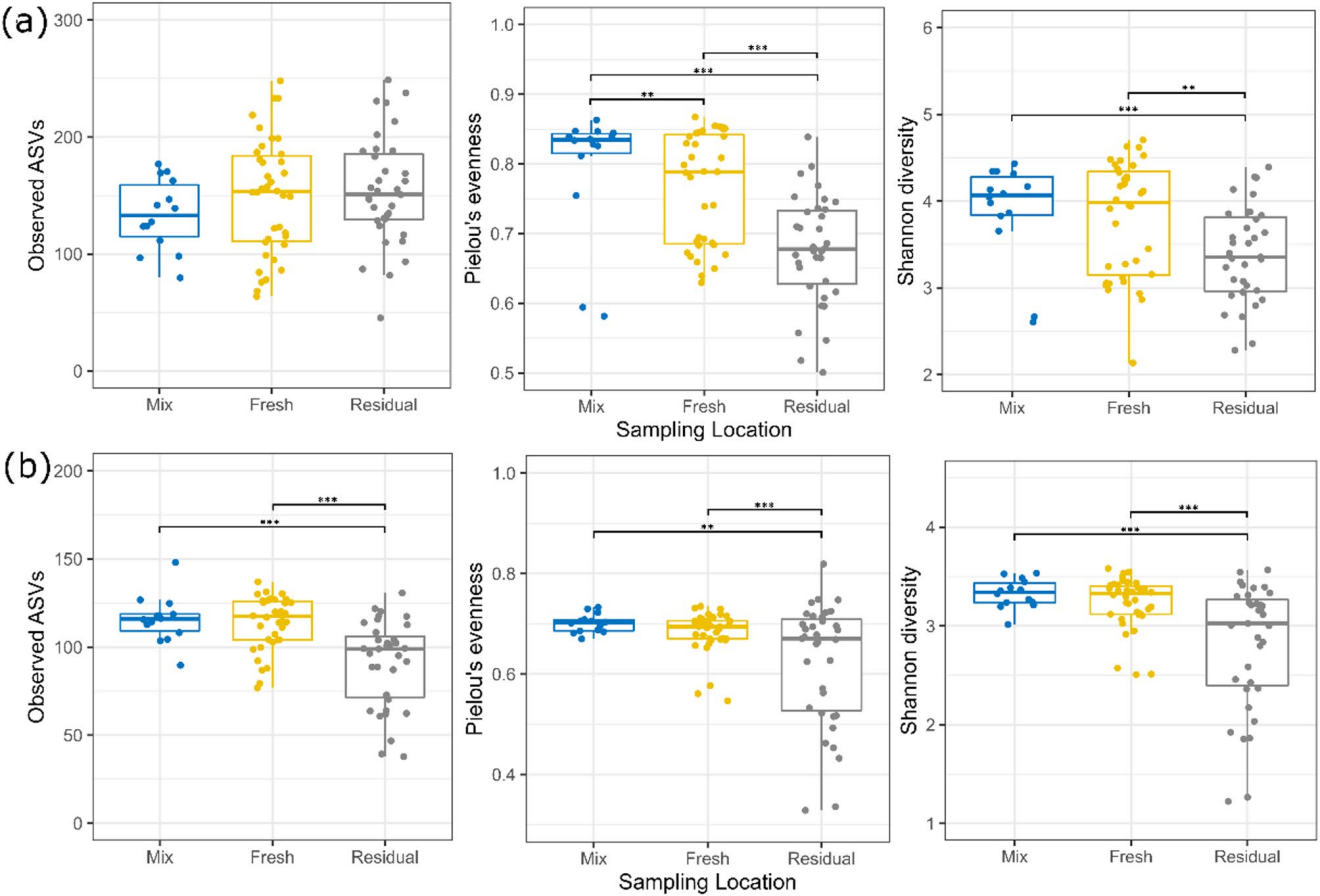
Boxplots displaying observed amplicon sequence variants (ASVs), Pielou’s evenness and Shannon diversity of bacterial (**a**) and fungal (**b**) communities in liquid feed samples from the mixing tank and in fresh and residual liquid feed samples from troughs on eight commercial pig farms. ** *p* ≤ 0.01, *** *p* ≤ 0.001. Mixing tank (Mix): *n* = 14, Fresh trough (Fresh): *n* = 38, Residual trough (Residual): *n* = 35.

The alpha-diversity of the bacterial and fungal communities in the liquid feed samples from the mixing tank and in the fresh and residual liquid feed samples from the troughs across the commercial pig farms is summarised in Fig. 1A and B, respectively. The number of observed bacterial ASVs did not differ between sampling locations (*p* > 0.05), indicating that bacterial species richness (number of species present) was similar. However, Pielou’s evenness (distribution of abundances, with high evenness indicating that all species are present in similar proportions) and Shannon diversity (measure of both richness and evenness) both decreased between the mixing tank and residual trough samples (*p* ≤ 0.001) and between the fresh and residual trough samples (Pielou’s evenness: *p* ≤ 0.001; Shannon diversity: *p* ≤ 0.01). Evenness also decreased between the mixing tank and fresh trough feed (*p* ≤ 0.01). The number of observed ASVs assigned to fungi decreased both between the mixing tank and the residual trough feed (*p* ≤ 0.001) and between the fresh trough and the residual trough feed (*p* ≤ 0.001), indicating that species richness decreased across sampling locations. Pielou’s evenness and Shannon diversity of the fungal communities also decreased from the mixing tank (Pielou’s evenness: *p* ≤ 0.01; Shannon diversity: ≤ 0.001) and fresh trough feed (Pielou’s evenness and Shannon diversity: *p* ≤ 0.001) to the residual trough feed.

Supplementary Figures S1–S6 show the bacterial and fungal alpha-diversity of the liquid feed sampled on each of the farms at each of the sampling locations. The liquid feed sampled from Farm H (research unit) had numerically higher bacterial alpha-diversity values than the other farms. To determine if the high microbial diversity on Farm H was the driver of the differences observed across all farms, differences in bacterial and fungal alpha-diversity between sampling locations on Farms A-G only was investigated (Supplementary Fig. S7). The results were generally in agreement with those obtained when Farm H data were included in the analysis, with significant decreases in alpha-diversity observed between mixing the feed and sampling from the troughs. One notable difference when Farm H data were excluded was that the observed bacterial ASVs increased between the fresh and residual feed (*p* ≤ 0.05), whereas this was not the case when Farm H data were included.

**Fig. 2:**
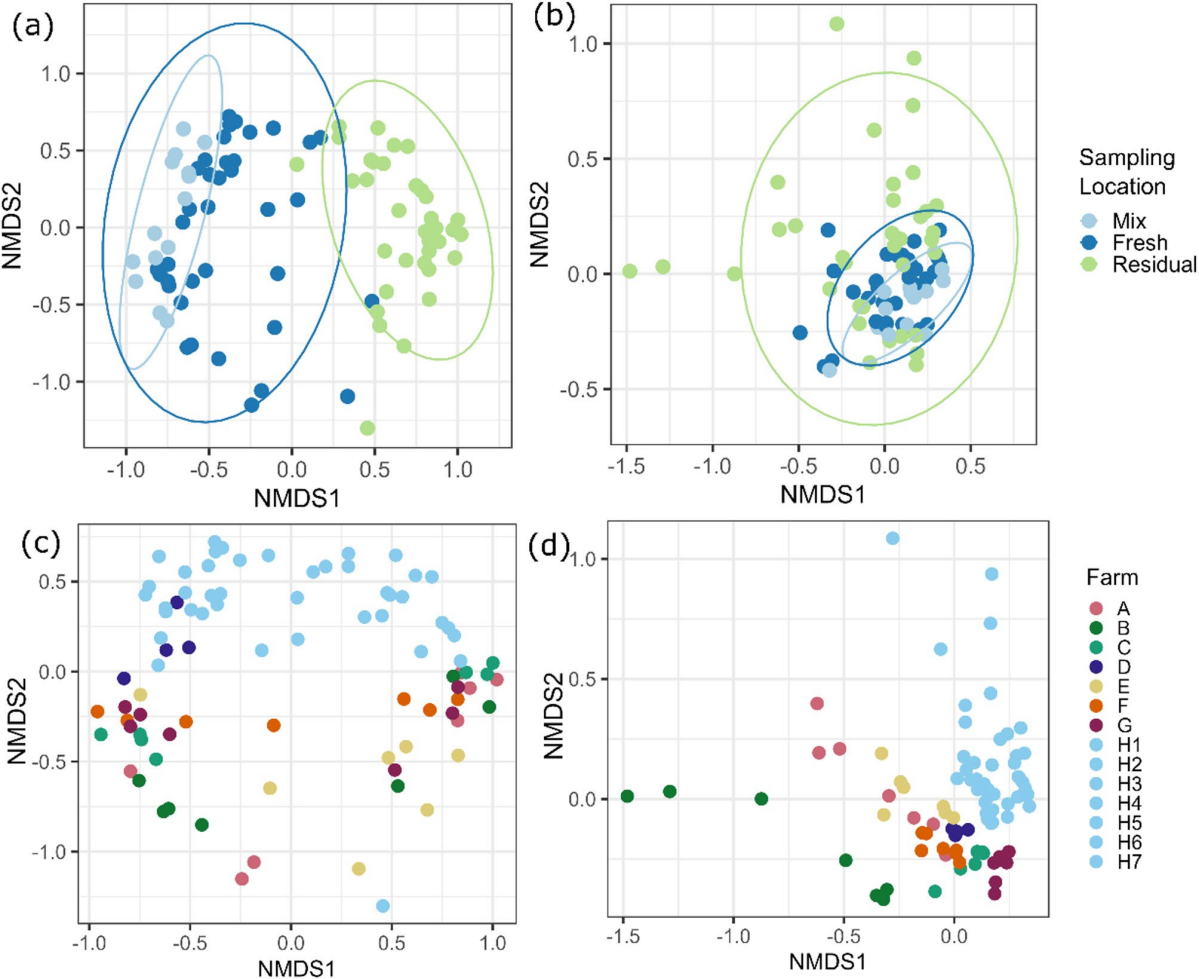
Non-metric multidimensional scaling (NMDS) plots based on Bray–Curtis dissimilarity in liquid feed collected at each respective sampling location; Mixing tank (Mix; *n* = 1/farm), liquid feed sampled immediately after delivery to the troughs (Fresh; *n* = 3/farm), liquid feed sampled prior to the next feed (Residual; *n* = 3/farm) on eight commercial pig farms for bacterial (**a**) and fungal (**b**) communities, and between farms for bacterial (**c**) and fungal (**d**) communities. Farms A–G were sampled on one sampling occasion, while Farm H was sampled on seven different occasions (H1–H7).

The NMDS plots indicated significant differences in microbial community structure between sampling locations, with clustering observed for both the bacteriome and mycobiome at each sampling location [Fig. 2A and B], respectively. The bacteriome in the mixing tank feed was more similar to that of the fresh liquid feed, while the residual samples were more dissimilar. The mycobiome was less dissimilar between sampling locations compared to the bacteriome, and the residual feed had greater variability between samples, as indicated by greater distances between points. Samples from the same farm generally clustered together, with the mycobiome generally being more similar between farms, compared to the bacteriome, as indicated by a greater degree of clustering [Fig. 2C and D]. In addition, as observed across the entire dataset, within-farm samples also clustered based on sampling location. Notably, the majority of samples from the different sampling occasions on Farm H (research farm) formed a distinct cluster away from the other farms with respect to both bacterial and fungal communities. For this reason, NMDS plots of Bray–Curtis dissimilarities between sampling location and farm were also prepared without data from the Farm H samples (Supplementary Fig. S8). These showed that the bacteriome and mycobiome of liquid feed from Farms A-G still formed distinct clusters based on sampling location and farm.

Permutational analysis of variance revealed that the variable ‘farm’ explained ~ 58% (*p* < 0.001) of the variation in bacterial community structure, while ‘sampling location’ (mixing tank vs fresh vs residual trough samples) was responsible for ~ 17% (*p* < 0.001). The variation in the fungal community structure was also influenced by farm, which explained ~ 61% (*p* < 0.001) of variation, with sampling location accounting for ~ 9% (*p* < 0.001) of variation. Due to the distinct clustering of the Farm H samples, PERMANOVA was also performed without the Farm H sample data in order to assess whether the farm variable effect was mainly driven by Farm H. This analysis confirmed that on Farms A-G, bacterial and fungal community structure was still significantly influenced by both farm (~ 67% and ~ 66% of variation, respectively; *p* < 0.001) and sampling location (~ 12% and ~ 8% of variation, respectively; *p* < 0.001).

### Comparison of bacterial and fungal taxa in liquid feed between sampling locations

**Fig. 3:**
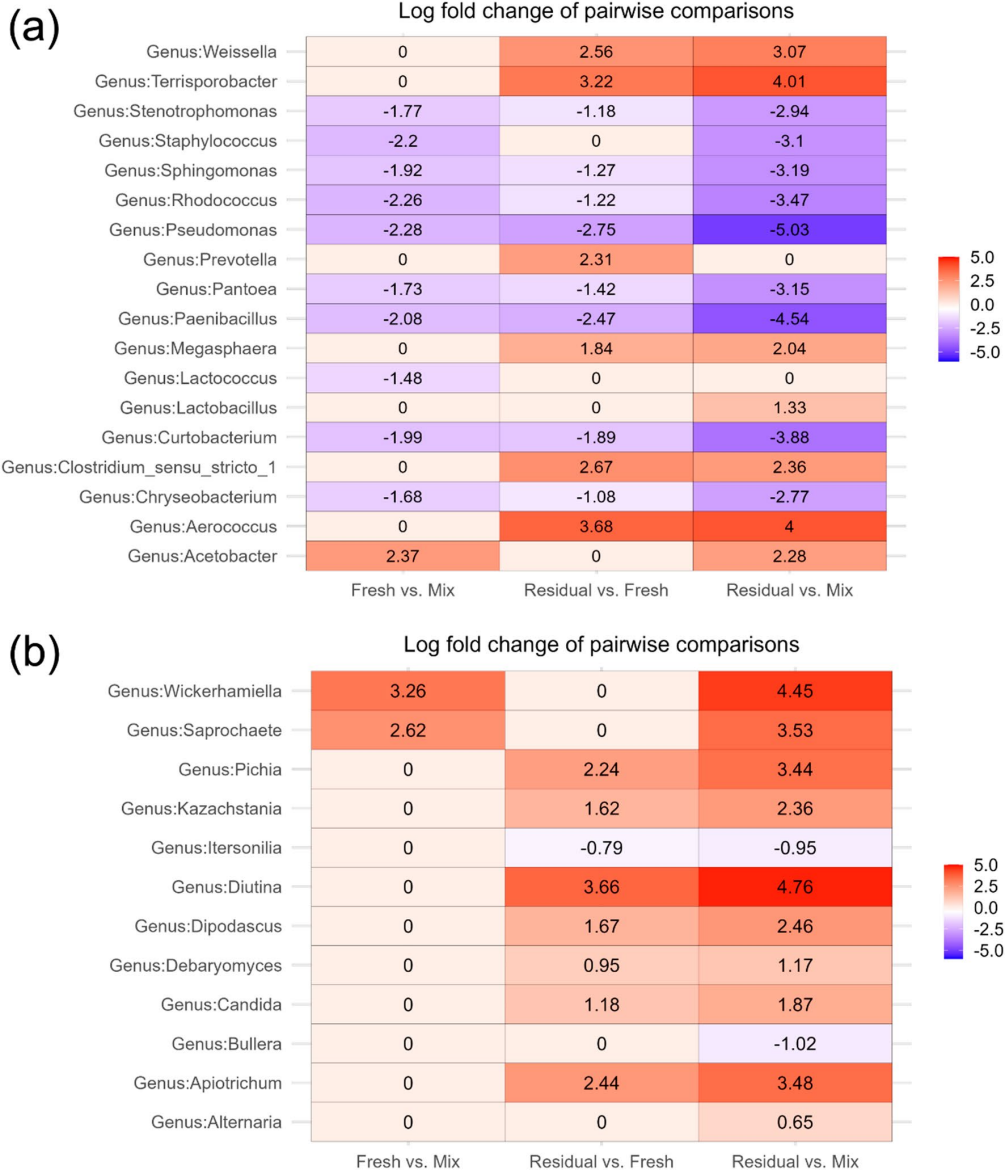
Heatmaps of log-fold changes of differentially abundant bacterial (**a**) and fungal (**b**) genera after pairwise comparisons between different liquid feed sampling locations. Log-fold changes of taxa between sampling locations with an adjusted *p*-value ≤ 0.05 were considered significant.

The relative abundance (RA) of bacterial and fungal taxa in liquid feed, averaged across all farms at the phylum-, family- and genus-level are available in Supplementary Data S1. The mean RA of the top 25 most abundant bacterial and fungal genera at each sampling location are summarised in Supplementary Fig. S9. In terms of the bacteriome of the residual trough-sampled feed, compared to that of the mixing tank, the LAB *Weissella* and *Lactobacillus* were enriched, along with *Terrisporobacter, Clostridium* sensu stricto 1 and *Aerococcus* (*p* < 0.05) [Fig. 3A]*.* The majority of these genera also increased in abundance between the fresh and residual feed, while *Acetobacter* was enriched in the fresh trough-sampled feed compared to the mixing tank. Bacterial genera that decreased in abundance in the troughs compared to the mixing tank, included *Pseudomonas, Sphingomonas* and *Pantoea* (*p* < 0.05). The majority of the fungi found to be differentially abundant between the sampling locations were yeasts; all of which were enriched between the mixing tank and residual feed, except *Bullera* which decreased (*p* < 0.05) [Fig. 3B]. *Wickerhamiella, Diutina* and *Apiotrichum* had the highest log-fold increases between the mixing tank and residual trough-sampled feed (*p* < 0.05). Two genera of filamentous basidiomycetes were differentially abundant (*p* < 0.05); *Itersonilia* decreased in the residual feed compared to the mixing tank and fresh trough-sampled feed, while *Alternaria* increased.

### Comparison of bacterial and fungal taxa in liquid feed between farms

**Fig. 4:**
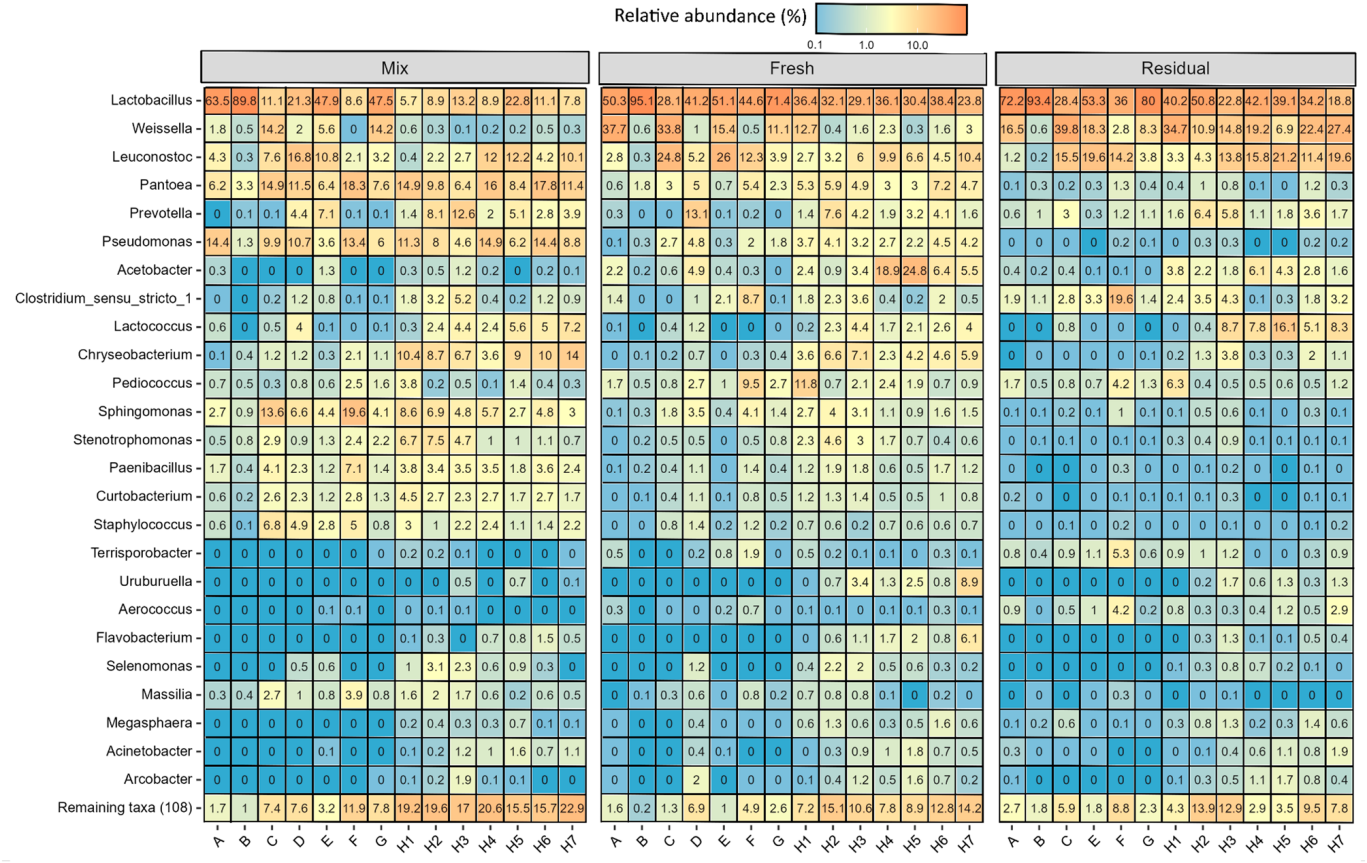
Heatmap displaying relative abundance (%) of the 25 most abundant bacterial genera in liquid feed sampled on each farm from the mixing tank (Mix: *n* = 1/farm), and from the troughs, immediately after delivery (Fresh: *n* = 3/farm*) and just before the next feed-out (Residual: *n* = 3/farm*). *No residual trough samples were collected on Farm D. On four of the seven sampling occasions on Farm H, only two fresh and residual trough samples were collected.

The RA of bacterial and fungal taxa at the phylum-, family- and genus-levels at each sampling location on an individual farm basis are available in Supplementary Data S1. There was considerable farm-to-farm variation in the bacteriome found at each sampling location (Fig. 4). *Lactobacillus* was the predominant bacterial genus detected in the mixing tank feed on the majority of farms (13.2–89.8% RA), and was highest on Farm B. On the other hand, *Pantoea* was the most abundant genus in the mixing tank on Farms/Samples C, H1, H2, H4 and H6 (9.8–17.8% RA). Lastly, *Sphingomonas* (19.6% RA) and *Chryseobacterium* (14% RA) were the predominant bacterial taxa in the mixing tank feed on Farm F and Sample H7, respectively. In the fresh liquid feed sampled from the troughs, *Lactobacillus* became the predominant genus on all farms (23.8 ± 1.5–95.1 ± 0.6% RA), except on Farm C, where it was *Weissella* (33.8 ± 2.0%). *Lactobacillus* remained predominant in the residual trough-sampled feed on most farms. However, *Weissella* still had the highest RA on Farm C, and also became the most abundant genus in the residual feed H7. In addition, the finding that samples from the research farm (H) had higher bacterial alpha-diversity compared to the other farms is supported by the fact that ~ 20% of the total bacterial reads in Farm H mixing tank samples were from genera other than the 25 most abundant (Fig. 4.). Also of note is the high RA of *Acetobacter* in the fresh trough feed on Farm H, but only on two sampling occasions (H4 and H5) and *Pediococcus* on one occasion (H1), highlighting the variation across sampling time points. Finally, *Clostridium* sensu stricto 1 was more prevalent on Farm F (RA of 19.6 ± 5.5% in the residual trough feed, compared to < 5% on all other farms).

**Fig. 5:**
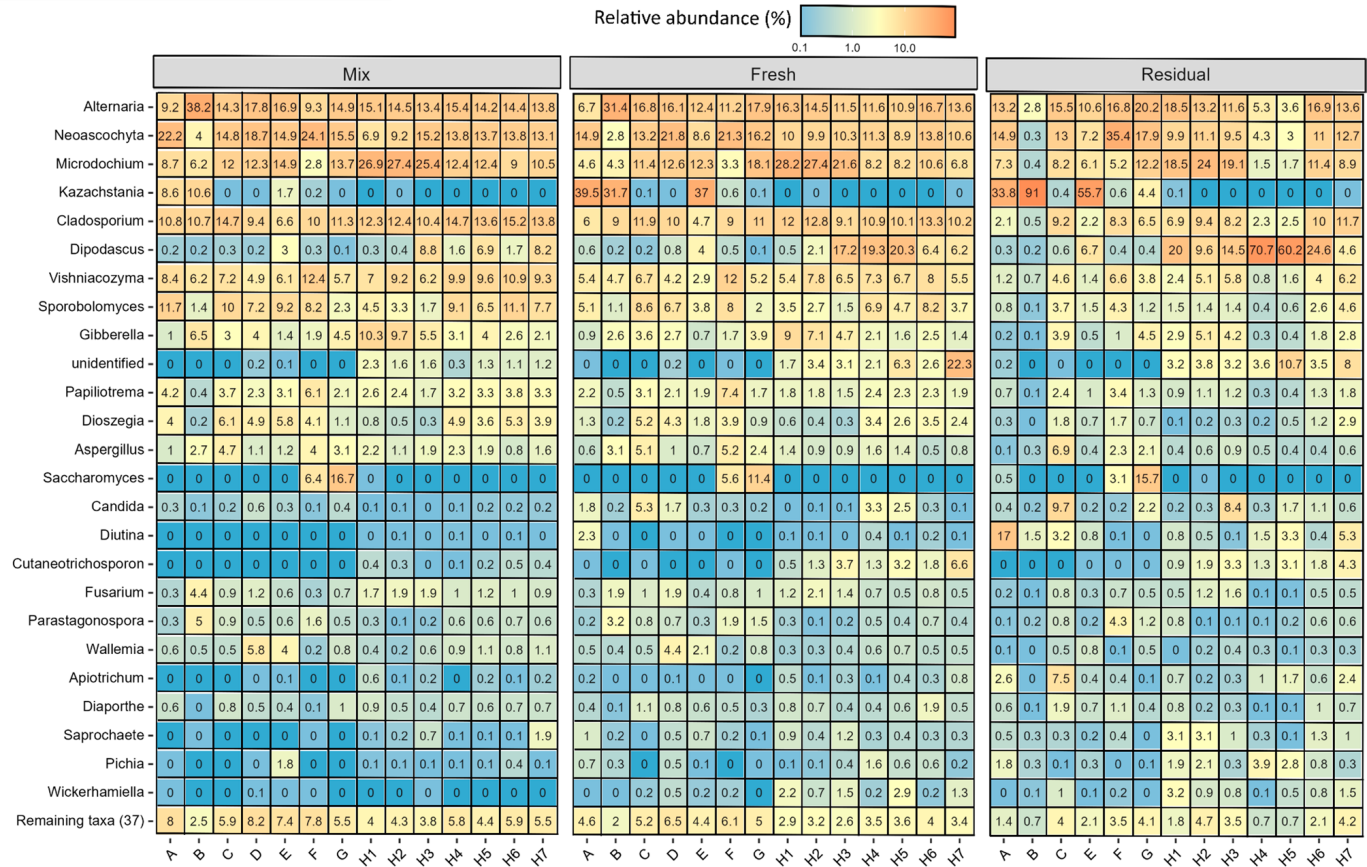
Heatmap displaying relative abundance (%) of the top 25 most abundant fungal genera in liquid feed on each farm in the mixing tank (Mix: *n* = 1/farm), and from the troughs, immediately after delivery (Fresh: *n* = 3/farm*) and just before the next feed-out (Residual: *n* = 3/farm*). *No residual trough samples were collected on Farm D. On four of the seven sampling occasions on Farm H, only two fresh and residual trough samples were collected.

The mycobiome of the mixing tank feed also varied across farms (Fig. 5). *Neoascochyta* was most abundant in the mixing tank feed of farms A, C, D and F (14.8–24.1% RA). *Alternaria* predominated on Farms B and E and in the H4 and H5 samples (14.2–38.2% RA), while the yeast, *Saccharomyces* predominated on Farm G (16.7% RA). In fact, the only farms on which *Saccharomyces* was detected in the mixing tank were Farms G and F. As with the bacteriome, the most abundant fungal genus in the mixing tank feed varied across time points on Farm H, with either *Microdochium* or *Cladosporium* predominating. Two yeast genera became dominant in the fresh trough-sampled feed on some farms; *Kazachstania* was most abundant on Farms A, B and E (31.7 ± 2.2–39.5 ± 8.1% RA), while *Dipodascus* predominated in the H4 (19.3 ± 7.1% RA) and H5 (20.3 ± 2.5% RA) samples. Meanwhile, cereal-associated filamentous fungi predominated in the fresh liquid feed on the remaining farms; *Alternaria* on Farm C and in the H6 and H7 samples (13.6 ± 0.7–16.8 ± 1.3% RA); *Microdochium* on Farm G and in the H1, H2 and H3 samples (18.1 ± 1.6–28.2 ± 1.5% RA); and *Neoascochyta* on Farms D (21.8 ± 0.7% RA) and F (21.3 ± 0.4% RA).

Between the fresh and residual trough-sampled feed, the predominant fungal genera remained the same on the majority of the farms/samples from the same farm. Some of the farms where yeasts were already predominant in the fresh feed, showed further increases in the residual feed; *Kazachstania* increased up to 55.7 ± 3.0% and 91 ± 4.2% RA on Farms E and B, respectively. Similarly, *Dipodascus* increased from 19.3 ± 7.1 to 70.7 ± 6.4% RA and from 20.3 ± 2.5 to 60.2 ± 17.8% RA in the H4 and H5 samples, respectively. *Neoascochyta* became the most abundant genus in the residual feed on Farm G (17.9 ± 0.6% RA), while *Dipodascus* predominated in the H1 (20.0 ± 11.9% RA) and H6 (24.6 ± 2.6% RA) samples. Interestingly, as for the mixing tank samples, *Saccharomyces* was only detected at > 1% RA in the troughs on Farms F and G.

### Biogenic amine concentrations in liquid feed

**Fig. 6:**
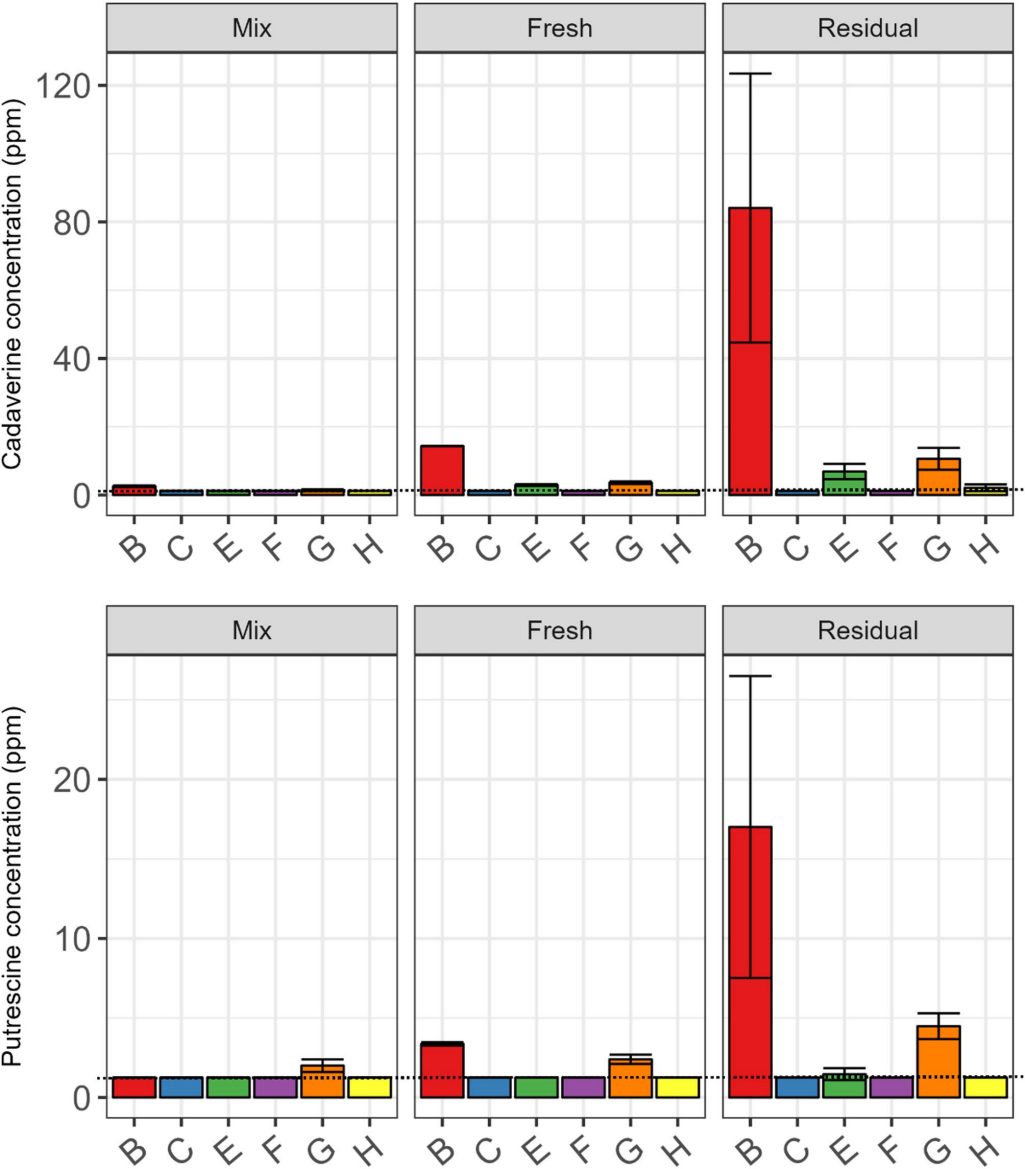
Biogenic amine concentrations in liquid feed sampled from mixing tanks (Mix) and troughs (Fresh and Residual) on six commercial pig farms. Biogenic amine concentrations are presented as ppm, on a fresh matter basis i.e. in the liquid sample. The limit of detection (LOD; dotted horizontal line on plots) for putrescine and cadaverine was 1.25 ppm and for tryptamine, histamine and tyramine it was 2.5 ppm; values below the LOD were recorded as being at the LOD. For the mixing tank, values are the mean of duplicate extractions of one sample (*n* = 1) and for fresh and residual trough-sampled feed, values are the mean of duplicate extractions of two samples (*n* = 2) except for the fresh trough samples for Farm B where values are the mean of duplicate extracts from 1 sample (*n* = 1). Standard deviations of duplicate extractions per sample are indicated by error bars.

Cadaverine and putrescine concentrations in the liquid feed sampled from the mixing tank and troughs are shown in Fig. 6, as these were the biogenic amines detected at the highest concentrations in the feed. Even for these, concentrations were generally low/non-detectable across farms, except for Farms B and G. On these farms, putrescine and cadaverine concentrations were highest in the residual trough-sampled feed, with Farm B having the highest concentrations. As regards the other biogenic amines analysed; histamine and tyramine were non-detectable on all farms; the only samples in which tryptamine was detected were the residual trough samples on Farm B; and Farm E had cadaverine concentrations of ~ 3 ppm in fresh trough samples, which increased to just below 7 ppm in the residual samples.

### Correlation of differentially abundant genera with biogenic amine, lactate, ethanol and VFA concentrations in liquid feed

**Fig. 7:**
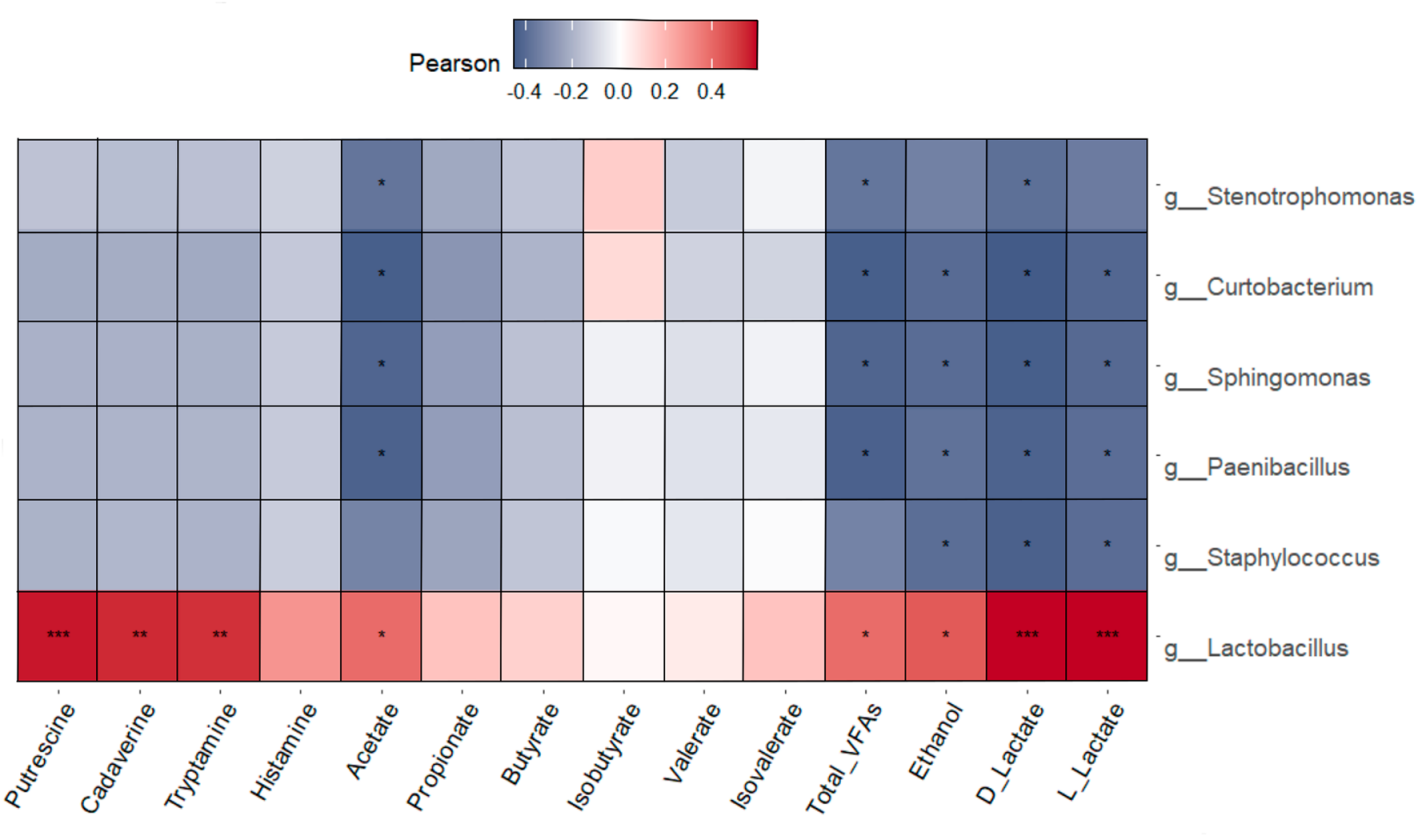
Pearson correlation plot of differentially abundant bacterial genera found in the liquid feed samples against biogenic amine, volatile fatty acid, ethanol and lactate concentrations in the liquid feed. * *p* ≤ 0.05, ** *p* ≤ 0.01, *** *p* ≤ 0.001. False discovery rate multiple testing correction was performed. Red indicates a positive correlation, white indicates no correlation and blue indicates a negative correlation.

**Fig. 8:**
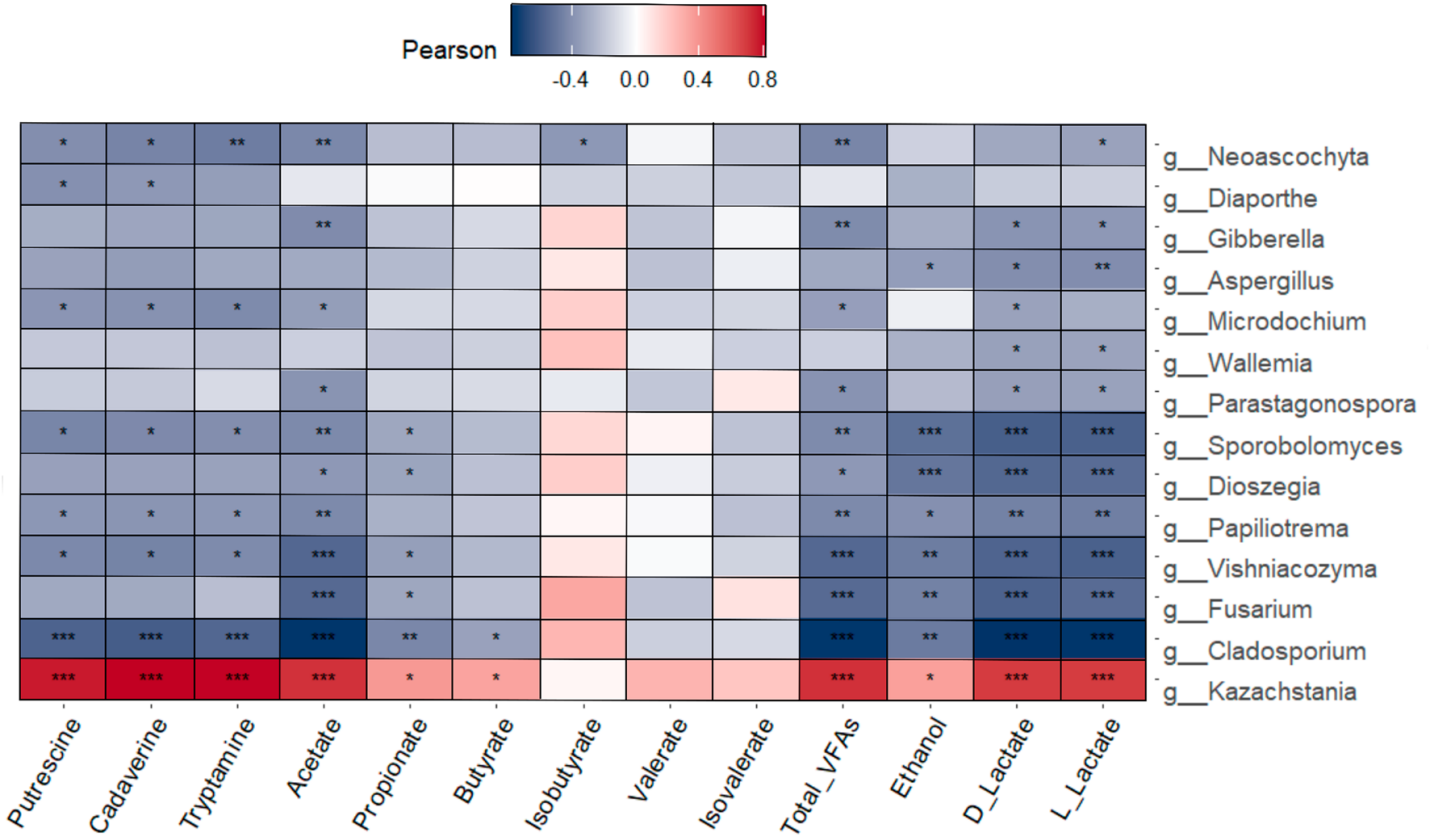
Pearson correlation plot of differentially abundant fungal genera found in the liquid feed samples against biogenic amine, volatile fatty acid, ethanol and lactate concentrations in the liquid feed. * *p* ≤ 0.05, ** *p* ≤ 0.01, *** *p* ≤ 0.001. False discovery rate multiple testing correction was performed. Red indicates a positive correlation, white indicates no correlation and blue indicates a negative correlation.

Correlations between differentially abundant microbial taxa and biogenic amine, VFA, ethanol and lactate concentrations in the same liquid feed samples are presented in Figs. 7 and 8 for bacterial and fungal genera, respectively. *Lactobacillus* and the yeast *Kazachstania* were the only taxa that had positive associations with these microbial metabolites in the liquid feed. *Lactobacillus* was positively correlated with putrescine (*r* = 0.57, *p* ≤ 0.001), cadaverine (*r* = 0.54, *p* ≤ 0.01) and tryptamine (*r* = 0.52, *p* ≤ 0.01), as well as with D-lactate and L-lactate (*r* = 0.59, *p* ≤ 0.001). *Lactobacillus* abundance was also positively associated with acetate (*r* = 0.39, *p* ≤ 0.05), total VFAs (*r* = 0.39, *p* ≤ 0.05) and ethanol (*r* = 0.43, *p* ≤ 0.05). *Kazachstania* was positively correlated with putrescine (*r* = 0.77, *p* ≤ 0.001), cadaverine (*r* = 0.81, *p* ≤ 0.001) and tryptamine (*r* = 0.80, *p* ≤ 0.001), as well as with D-lactate and L-lactate (*r* = 0.69, *p* ≤ 0.001). Positive associations were also found between *Kazachstania* and acetate (*r* = 0.72, *p* ≤ 0.001), propionate (*r* = 0.37, *p* ≤ 0.05), butyrate (*r* = 0.34, *p* ≤ 0.05), total VFAs (*r* = 0.72, *p* ≤ 0.001) and ethanol (*r* = 0.35, *p* ≤ 0.05).

The abundances of a number of bacterial and fungal taxa were negatively correlated with concentrations of certain feed metabolites. *Stenotrophomonas*, *Curtobacterium, Sphingomonas* and *Paenibacillus* were all negatively associated with acetate (*p* ≤ 0.05), total VFAs (*p* ≤ 0.05), ethanol (*p* ≤ 0.05) and D- and L-lactate (*p* ≤ 0.05), while *Staphylococcus* was negatively correlated with ethanol (*r* = − 0.40, *p* ≤ 0.05), D-lactate (*r* = 0.43, *p* ≤ 0.05) and L-lactate (*r* = 0.40, *p* ≤ 0.05). As regards fungi, *Cladosporium* had the greatest number of negative correlations, along with the highest correlation coefficients. It was negatively associated with the biogenic amines putrescine (*r* = − 0.59, *p* ≤ 0.001), cadaverine (*r* = − 0.61, *p* ≤ 0.001) and tryptamine (*r* = − 0.57, *p* ≤ 0.001). *Cladosporium* was also negatively correlated with acetate (*r* = − 0.77, *p* ≤ 0.001), propionate (*r* = − 0.45, *p* ≤ 0.01), butyrate (*r* = − 0.34, *p* ≤ 0.05) and total VFAs (*r* = − 0.76, *p* ≤ 0.001), in addition to ethanol (*r* = − 0.49, *p* ≤ 0.01), D-lactate (*r* = − 0.78, *p* ≤ 0.001) and L-lactate (*r* = − 0.77, *p* ≤ 0.001).

## Discussion

This study profiled bacterial and fungal communities and biogenic amine concentrations of liquid feed for grow-finisher pigs on a selection of commercial pig farms. Differentially abundant microbial taxa were correlated with microbial metabolite (biogenic amine, VFA, ethanol and lactate) concentrations in the same liquid feed samples. Despite its widespread adoption, there are a number of disadvantages of liquid feeding, most notably the poorer FCE observed in liquid-fed pigs^4,5,32,33^. One potential reason for this is the unintentional spontaneous fermentation observed in liquid feed which can lead to depletion of dietary energy and amino acids, as well as production of undesirable concentrations of ethanol and acetic acid^2,3,5,6,34–37^. Liquid feed provides a source of fermentable carbohydrate for LAB and yeast, and hence, the occurrence of LAB and yeast fermentation in liquid feed is well-documented^2,5,34,38,39^. However, many studies to date have focused on the microbial communities of deliberately fermented liquid feed, while the farms in the current study fed ‘fresh’ liquid feed i.e. not deliberately fermented. Additionally, since most studies on liquid feed microbiota have used only culture-based methods, this study is fundamental in establishing the complete bacterial and fungal profile of fresh liquid feed using culture-independent methods, and in determining the extent of biogenic amine production on commercial pig farms. Additionally, it correlates, for the first time, specific microbial taxa with microbial metabolites in liquid feed.

Decreases in alpha-diversity of the bacterial and fungal communities in the feed were found between the mixing tank and the troughs. In addition, there was distinct clustering of the bacteriome and mycobiome at each sampling location, in agreement with the fact that sampling location was a significant determinant of bacterial and fungal composition. The decreased microbial diversity in the feed after mixing is likely a result of spontaneous fermentation by LAB and yeast, as reported by O’Meara et al.^3^ on these farms. This was evidenced in the same samples as those analysed in the present study by decreased pH and increased LAB and yeast counts in trough-sampled feed compared with the mixing tank, as well as amino acid and energy losses^3^. The compositional data from the present study confirm this, with the LAB *Lactobacillus, Weissella* and *Leuconostoc* predominating in the feed, and *Lactobacillus* and *Weissella* increasing sequentially in abundance from the mixing tank to the freshly delivered feed in the troughs to the residual feed sampled just before the next feed-out. Positive correlations between *Lactobacillus* and *Kazachstania* and biogenic amine, VFA, ethanol and lactate concentrations in the liquid feed further support the participation of these taxa in spontaneous fermentation. A concurrent decrease in the RA of Gram-negative bacteria including *Pantoea* and *Pseudomonas* occurred. This is in line with the second phase of feed fermentation where low pH and increasing concentrations of lactic acid, and other metabolites, produced by LAB fermentation, inhibit pathogenic and spoilage bacteria^5,37,40^. Hence, this bacterial shift could be considered beneficial. However, this is dependent on whether the LAB are homofermentative or heterofermentative. The former is desirable as lactic acid is produced as the sole fermentation product, while the latter result in the production of lactic acid, CO_2_, ethanol and/or acetic acid^41^. Correlation of *Lactobacillus* with concentrations of lactate, acetate and ethanol suggests that a substantial proportion of the *Lactobacillus* population in the liquid feed may have been heterofermentative. However, species-level assignment is necessary to determine this and this was not achieved in the current study.

Microbial community structure in the feed was influenced mostly by the farm of origin, which explained ~ 60% of the variance between samples. This is perhaps unsurprising, as each surveyed farm had different diet compositions, liquid feeding systems and feeding practices. However, *Lactobacillus*, *Pantoea* and *Pseudomonas* were the most abundant bacterial genera in the mixing tanks, on average, across the farms. Furthermore, despite the differences in the mixing tank bacteriome between farms, once the feed was delivered to the troughs, LAB (primarily *Lactobacillus* but also *Weissella* and *Leuconostoc*) predominated across all farms. Variability of spontaneous fermentation, however, was also evident, with between-farm differences in the genera that predominated. For example, *Acetobacter,* which was < 1% RA in the H4 and H5 mixing tank samples, increased to reach 18.9 ± 5.7 and 24.8 ± 12.4% RA, respectively, in the fresh trough-sampled feed. However, in the troughs, LAB appeared to outcompete *Acetobacter,* which fell to 6.1 ± 3.5 and 4.3 ± 2.4% RA in the H4 and H5 residual feed samples, respectively. The residual feed from Farm F was also notable because *Clostridium* sensu stricto 1 occurred at a RA of ~ 20%. Clostridial fermentation is undesirable, potentially resulting in excessive butyric acid, ammonia and biogenic amine production, leading to feed palatability and health concerns^42^. However, biogenic amines were not detected in the feed from Farm F. Although the reason for the high proportion of *Clostridium* is unclear, the dietary inclusion of pot ale syrup and the lack of cleaning of the liquid feeding system on this farm may have contributed. Additionally, this farm operated a short trough system which can allow residual feed to remain in the troughs for longer, facilitating more feed fermentation^3^. The differences between farms appear to be determined by the initial microbiota of the feed. However, once the feed is mixed, a similar pattern occurs; the mixing tank and freshly sampled trough liquid feed remain relatively similar in composition, while the residual trough-sampled feed differs substantially.

Detection of biogenic amines in the liquid feed in the present study, particularly when residual feed remained in the troughs between feeds, provides further evidence of uncontrolled spontaneous fermentation. However, only two farms had appreciable concentrations. The presence of biogenic amines was not surprising as O’Meara et al. (unpublished) found a 35.6% loss of lysine between mixing tank- and residual trough-sampled feed, on average, across the 8 farms. Farm B, which had the highest concentrations of putrescine and cadaverine had a 34.8% loss (O’Meara et al., unpublished). The concentrations of putrescine and cadaverine in residual trough-sampled feed on this farm (~ 17 and ~ 84 ppm, respectively on a fresh matter basis) were higher than the average concentrations detected in a survey of French finisher pig liquid feed (7 and 57 ppm, respectively, although these were on a DM basis)^8^. However, they were lower than the maximum concentrations detected in that study (310 and 1182 ppm, respectively)^8^. Putrescine concentrations were similar to those observed in residual trough- sampled liquid feed by Torres-Pitarch et al.^43^ (27.8 ppm on a fresh matter basis) but cadaverine concentrations were higher (~ 84 ppm versus 18.5 ppm detected by Torres-Pitarch et al.^43^). However, it should be noted that there was considerable variation between the residual trough samples collected on Farm B (as indicated by the large standard deviation), likely resulting from varying degrees of fermentation in individual troughs. In contrast to Farm B, biogenic amine concentrations on Farm G were lower than in the aforementioned studies. Apart from acting as an indicator of spontaneous fermentation and amino acid decarboxylation in liquid feed, biogenic amines may also have implications for pig health; however, no limits are available for liquid feed. Histamine, which was undetectable in this study, is the biogenic amine of greatest concern, with a 50 ppm limit in food set by the US Food and Drug Administration^44^. The European Food Safety Authority reported that there is insufficient information available to determine the concentrations of putrescine and cadaverine associated with adverse health effects^45^; however, concentrations > 440.75 and 255.45 ppm, respectively, are toxic to intestinal cells in vitro^46^*.* Therefore, it is unlikely that the concentrations observed in this study are of concern.

The French study found that putrescine and cadaverine concentrations were higher for farms using liquid co-products or pre-fermented high moisture corn in their diets (101–141 and 211–616 ppm on a DM basis, respectively)^8^. Interestingly, Farms F and G in the current study included pot ale syrup in their diets, which may have contributed to the higher concentrations of biogenic amines on Farm G compared to the other farms. Although little is known about the microbiology of pot ale syrup, it has a low pH and is dominated by yeast and lactobacilli^47^. Farm B, however, did not use liquid co-products in their diet; nonetheless, LAB and yeast counts in residual trough-sampled liquid feed were highest on Farm B (O’Meara et al., unpublished). This helps to explain the high biogenic amine concentrations found on this farm, particularly in the residual trough samples. Niven et al.^36^ demonstrated in vitro that *E. coli* was primarily responsible for lysine metabolism, and hence cadaverine formation, in liquid feed. However, numerous LAB strains including members of *Lactobacillus, Leuconostoc* and *Pediococcus* are well-known to produce biogenic amines in fermented foods^48^. Furthermore, there is evidence of amino acid decarboxylation by yeasts^49,50^. Despite this, research into the role of LAB and yeast in amino acid metabolism and resultant biogenic amine production in liquid feed is lacking. However, in the current study, both *Lactobacillus* and *Kazachstania* were positively correlated with concentrations of putrescine, cadaverine and tryptamine, supporting the hypothesis of their role in biogenic amine formation in liquid feed.

Information regarding fungal communities in fresh liquid feed is relatively scarce, with the majority of studies to date focusing on culturing yeasts from deliberately fermented feed^51–56^, hence the value of the current study. For example, while O’Meara et al.^3^ reported that mould counts were similar across the same mixing tank and trough samples analysed in the current study, the culture-independent methods used here identified the prevalence of certain moulds and variations between sampling locations. Some of the moulds most commonly associated with mycotoxin production, including *Alternaria*, *Cladosporium*, *Aspergillus* and *Fusarium* were detected on all surveyed farms^57,58^. Although not measured in this study, the prevalence of these fungi justifies profiling the mycotoxin content of liquid feed on commercial pig farms in future studies. *Alternaria* and *Cladosporium* in particular, were highly abundant on many farms, especially in the mixing tank, with their RA generally decreasing in the troughs. Farm B, a farm that performed no cleaning of the liquid feeding system^3^, had a particularly high prevalence of moulds; the mixing tank feed contained *Alternaria, Cladosporium*, *Aspergillus* and *Fusarium* at 38.2, 10.7, 2.7 and 4.4% RA, respectively. However, between the mixing tank feed and the fresh feed in the troughs, the yeast *Kazachstania* increased to reach 31.7 ± 2.2% RA, and subsequently almost completely dominated the residual feed (91 ± 4.2% RA). Yeast fermentation on Farm B was evidenced by an ethanol concentration of 24.8 mmol/kg in the residual trough-sampled feed (O’Meara et al., unpublished), compared to an average of 15.8 mmol/kg across all farms^3^. The predominance of *Kazachstania* in the residual trough-sampled feed on Farm B was likely due to its already high RA in the mixing tank feed (10.6%). *Kazachstania* was also present in the mixing tank feed of Farm A at high RA (8.6%). However, it was not as abundant in the residual feed compared to Farm B, likely due to competition from other yeasts including *Diutina* which was present at 17.0 ± 10.8% RA in the residual trough-sampled feed. Yeast-dominated fermentation is generally considered undesirable in liquid feed because excessive ethanol production may reduce feed palatability, and result in DM and energy losses from the diet^7,37,59^. Previous culture-based analysis of the samples from this study by O’Meara et al.^3^ provided evidence of yeast fermentation across all farms, with yeast counts and ethanol concentrations increasing between the mixing tank and the troughs. In general, in the present study, *Kazachstania* and *Dipodascus* were the most dominant yeasts across all farms, with the highest RA found in the residual feed, again indicating yeast fermentation. This was further supported by the positive correlation between *Kazachstania* and ethanol concentrations in the liquid feed. *Kazachstania* has previously been identified in deliberately fermented liquid feed^55,56^; however, to our knowledge *Dipodascus* has not been previously reported in liquid feed. The association of *Kazachstania* with biogenic amine concentrations in this study highlights another potential negative effect of excessive uncontrolled yeast fermentation in liquid feed. Interestingly, the farms that included pot ale syrup, a co-product from whiskey production, in their diets (Farms A, F and G) were the only farms where *Saccharomyces* was detected. Although the species was not identified, *Saccharomyces cerevisiae* is used in whiskey production and therefore, pot ale syrup is likely the source of these yeasts^60^. Furthermore, Farm A, which used liquid whey, a by-product of cheese-making, had the highest RA of *Diutina* in the residual feed. This yeast genus contains a number of species which are associated with dairy products, including cheese^61,62^. This highlights the impact that the initial microbial composition of the diet has on the subsequent progression of the bacteriome and mycobiome in the troughs.

## Conclusion

This study supports the current evidence that spontaneous fermentation occurs in ‘fresh’ liquid feed on commercial pig farms and provides insight into the bacterial and, for the first time, the fungal populations of liquid feed using high-throughput amplicon sequencing. The bacterial and fungal community structures in liquid feed were influenced, not only by the sampling location on a given farm (i.e. mixing tank versus troughs), but also by the farm from which the feed was sampled. This highlights the unpredictable nature and the between-farm variability of spontaneous fermentation. The dietary inclusion of liquid co-products influenced the microbial community in the liquid feed, with yeasts associated with co-products proliferating in the troughs. The decreases in alpha-diversity of the microbiota between the mixing tank and the troughs corresponded with increased RA of bacteria, particularly *Lactobacillus, Weissella* and *Leuconostoc,* as well as yeasts including *Kazachstania* and *Dipodascus*. Both *Lactobacillus* and *Kazachstania* were positively correlated with biogenic amine, VFA, ethanol and lactate concentrations in liquid feed. The concentration of biogenic amines also increased between the liquid feed in the mixing tank and the troughs, as a result of amino acid decarboxylation. While the biogenic amine concentrations observed in the current study are likely not of concern, the concomitant amino acid losses likely play a role in the poorer FE previously observed in liquid-fed pigs. Dietary acidification and improved feeding system hygiene could potentially limit spontaneous fermentation in fresh liquid feed and additional research is needed in these areas.

## Electronic supplementary material

Below is the link to the electronic supplementary material.

Supplementary Material 1

Supplementary Material 2

## Data Availability

Raw FASTQ files for this study have been deposited in the European Nucleotide Archive (ENA) at EMBL-EBI under project accession number PRJEB72728 (https://www.ebi.ac.uk/ena/browser/view/PRJEB72728).
