# Supplementary material for: Bacteriome and mycobiome profiling of liquid feed for finisher pigs on commercial pig farms
Source: Sci Rep. 2025 Jul 9;15:24718. doi: 10.1038/s41598-025-05928-8 (PMC12241619; doi:10.1038/s41598-025-05928-8)
Supplement: Supplementary file 1 — Supplementary Material 1 [file 41598_2025_5928_MOESM1_ESM.pdf]

Supplementary information for:

Bacteriome and mycobiome profiling of liquid feed for finisher pigs on commercial pig farms

J. T. Cullen[ab], P.G. Lawlor[b*], P. Cormican[c], F. Crispie[d] H. Slattery[d] and G.E. Gardiner[a]

[a] Eco-Innovation Research Centre, Department of Science, South East Technological University, Cork Road Campus, X91 K0EK County Waterford, Ireland

[b] Teagasc Pig Development Department, Animal and Grassland Research and Innovation Centre, Moorepark, Fermoy, P61 C996 County Cork, Ireland

[c] Animal and Bioscience Research Department, Animal and Grassland Research and Innovation Centre, Teagasc, Grange, Dunsany, C15 PW93 County Meath, Ireland.

[d] Teagasc Food Research Centre, Moorepark, Fermoy, P61 C996 County Cork, Ireland.

*Corresponding author: Peadar G. Lawlor. Email: Peadar.Lawlor@teagasc.ie

Supplementary Table S1**:** Summary of survey results on liquid feeding practices used in the finisher section on eight commercial pig production units (O'Meara et al. 2020a).

	Unit*							
	**A**	**B**	**C**	**D**	**E**	**F**	**G**	**H**†
Model of feed system	Funki	Funki	Funki	Funki	Datamix	BD‡	BD	BD
Trough type	Long	Long	Long§	Long	Short	Short	Short	Short
Age of feed system (yrs¶)	4	10	0.167	10	3	20	10	4
Feed splits (feeds/day)	5	4	4	4	10	12	4	4 or 5
Water to meal ratio (FM**)	2.1:1	2.4:1	3.5:1	3:1	2.6:1	3.4:1	3.1:1	2.5:1
Volume in mix tank (kg)	2,632	1529	11,000	1,500	3,000	3,000	11,000	~150
Agitation time (min)	23	20	10	2††	8 to 10	6††	30	5 to 20
Time for feed-out (min)	45	10	20	15	120	5	30	60
Pigs fed per trough	50	24	28	26	56	45	64	6
Pens on feed circuit	56	36	300	14	24	17	23	36
Pump method	Hydraulic	Hydraulic	Hydraulic	Hydraulic	Hydraulic	Hydraulic	Hydraulic	Pneumatic
Co-product inclusion	LW‡‡, PAS§§					PAS	PAS	

*All units were home milling, feeding at night-time (i.e. feeding between 18:00 and 06:00) and feeding equal percentages of the daily feed allowance at each feed time.

†Unit H: Research unit where feeds/day, volume in mixing tank, agitation time and time for feed-out varied depending on experimental criteria. This unit was investigated on seven occasions; whereas all other units were investigated on one occasion.

‡BigDutchman.

§Although this unit had a long trough feeding system, a large volume of feed was mixed and fed continuously over a period of time.

¶Years.

**On a fresh matter basis.

††Units D and F both had a satellite tank. Feed goes to the satellite tank after the mixing tank prior to being fed out. On unit D feed resided there for a very short period prior to feed out so the 2 min agitation is in the mixing tank. On unit F, agitation time was 3 min in the mixing tank and 3 min in the satellite tank.
‡‡Liquid whey.
§§Pot-ale syrup.

Supplementary Table S2: Dietary ingredients used in finisher diets on the eight pig production units surveyed (O'Meara et al. 2020a).

	**Pig Unit**							
	**A**	**B**	**C**	**D**	**E**	**F**	**G**	**H**
Barley	✓	✓	✓	✓	✓	✓	✓	✓
Wheat	✓	✓	✓	✓	✓			✓
Soya bean meal	✓	✓	✓	✓	✓	✓	✓	✓
Maize	✓			✓		✓	✓	
Oil (soya or not specified)	✓	✓	✓	✓	✓	✓	✓	✓
Soya hulls	✓		✓			✓	✓	
Minerals & vitamins	✓	✓	✓	✓	✓	✓	✓	✓
Pot ale syrup	✓[*]					✓[†]	✓[‡]	
Liquid whey	✓[§]							

[*] Inclusion rate 14%
[†] Inclusion rate 5%
[‡] Inclusion rate 10%
[§] Inclusion rate 21%

Supplementary Table S3: Concentration of individual biogenic amines (μg/mL) in standard mixes used for HPLC quantification of biogenic amines in liquid feed.

**Mixture No.**	**Histamine**	**Putrescine**	**Cadaverine**	**Tyramine**	**Tryptamine**
1	2.5	1.25	1.25	2.5	2.5
2	5	2.5	2.5	5	5
3	10	5	5	10	10
4	25	12.5	12.5	25	25
5	50	25	25	50	50

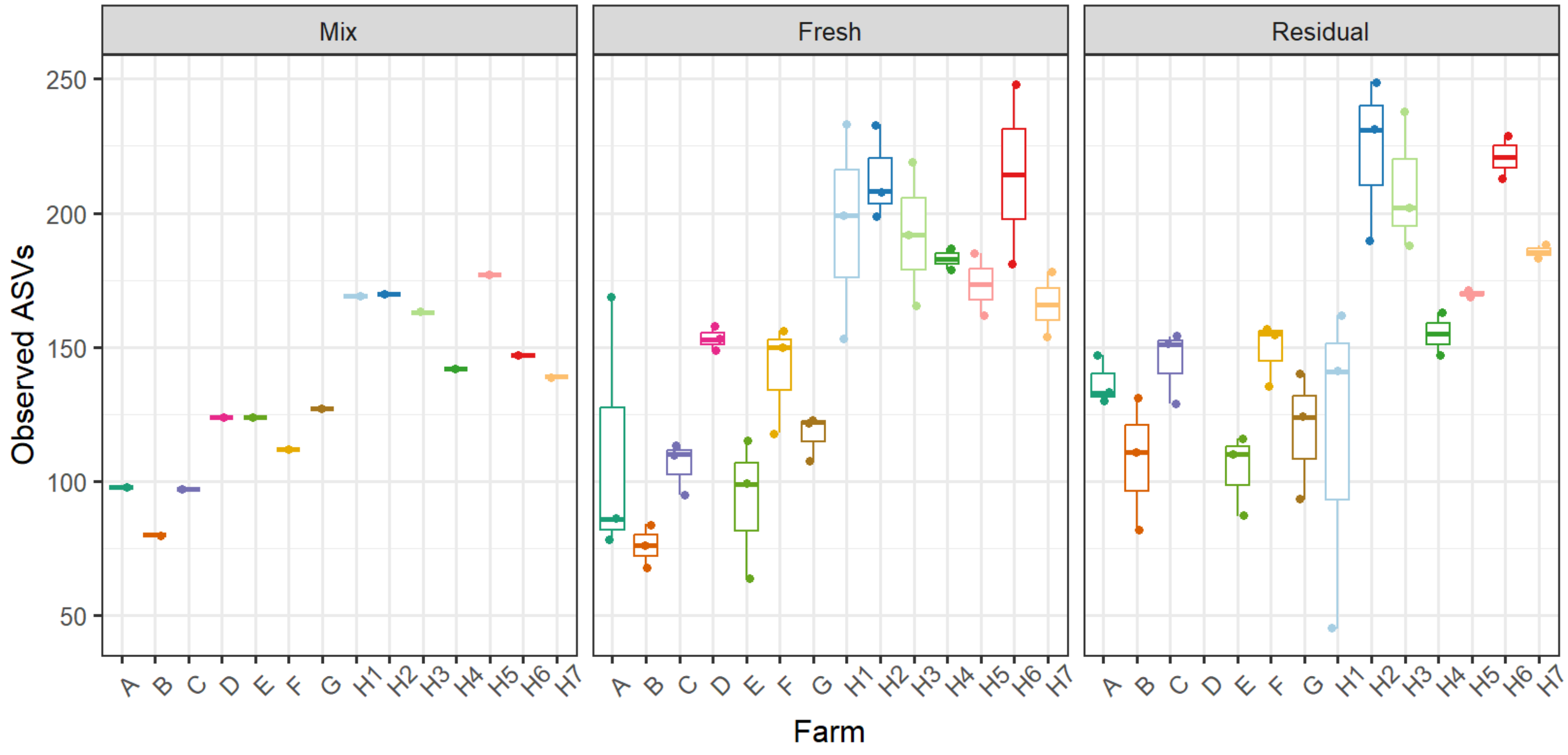

Supplementary Figure S1: Boxplots displaying observed amplicon sequence variants (ASVs) for bacterial taxa in liquid feed collected from each farm (A-H) at each respective sampling location; Mixing tank (Mix; *n* = 1), liquid feed sampled immediately after delivery to the troughs (Fresh; *n* = 3), liquid feed sampled prior to the next feed (Residual; *n* = 3). No Residual samples were collected on Farm D. H1-H7 indicate the seven occasions on which Farm H was sampled; on sampling occasions H4-H7 only two Fresh and two Residual samples were collected.

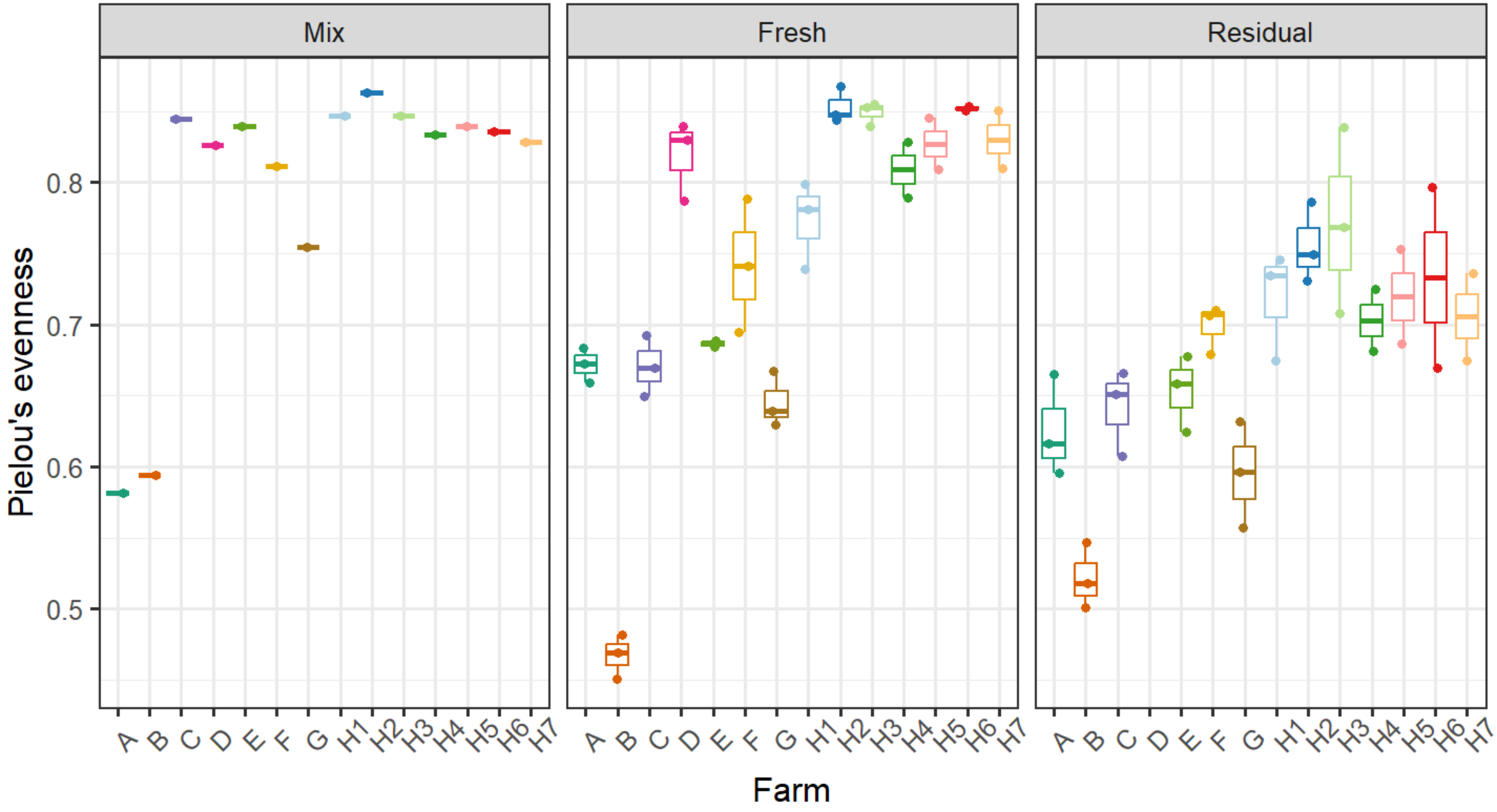

Supplementary Figure S2: Boxplots displaying Pielou's evenness of bacterial taxa in liquid feed collected from each farm (A-H) at each respective sampling location; Mixing tank (Mix; *n* = 1), liquid feed sampled immediately after delivery to the troughs (Fresh; *n* = 3), liquid feed sampled prior to the next feed (Residual; *n* = 3). No Residual samples were collected on Farm D. H1-H7 indicate the seven occasions on which Farm H was sampled; on sampling occasions H4-H7 only two Fresh and two Residual samples were collected.

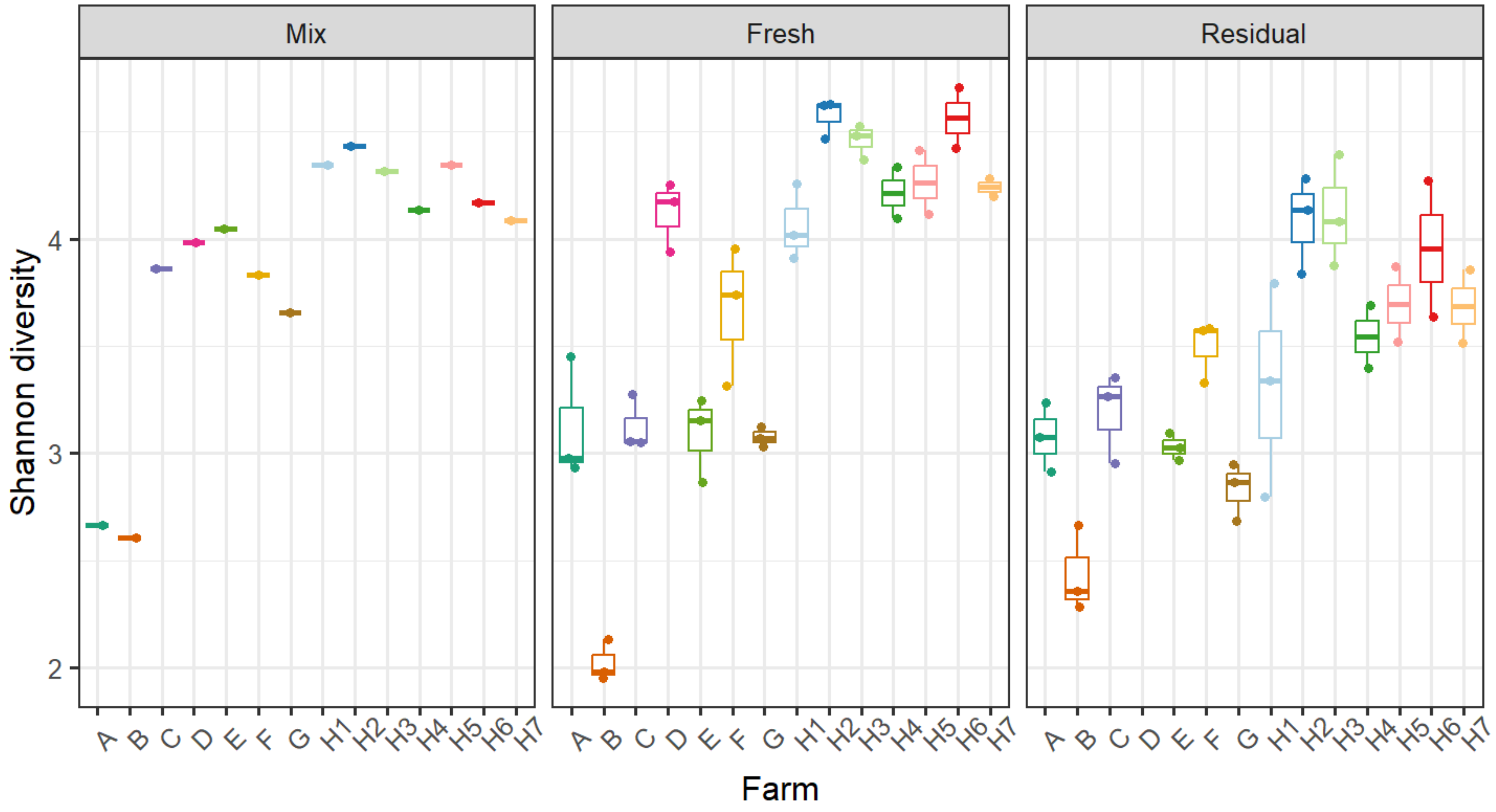

Supplementary Figure S3: Boxplots displaying Shannon diversity of bacterial taxa in liquid feed collected from each farm (A-H) at each respective sampling location; Mixing tank (Mix; *n* = 1), liquid feed sampled immediately after delivery to the troughs (Fresh; *n* = 3), liquid feed sampled prior to the next feed (Residual; *n* = 3). No Residual samples were collected on farm D. H1-H7 indicate the seven occasions on which Farm H was sampled; on sampling occasions H4-H7 only two Fresh and two Residual samples were collected.

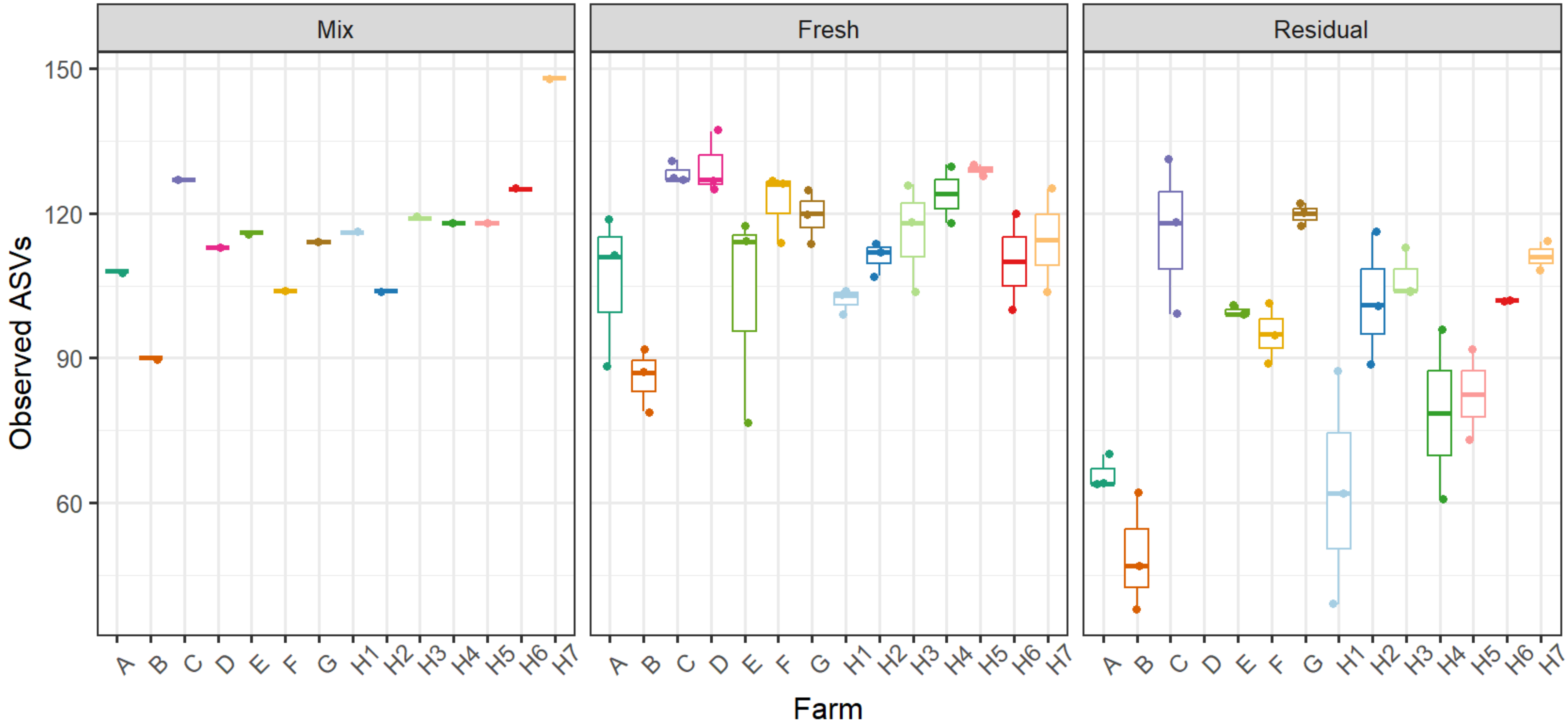

Supplementary Figure S4: Boxplots displaying observed amplicon sequence variants (ASVs) for fungal taxa in liquid feed collected from each farm (A-H) at each respective sampling location; Mixing tank (Mix; *n* = 1), liquid feed sampled immediately after delivery to the troughs (Fresh; *n* = 3), liquid feed sampled prior to the next feed (Residual; *n* = 3). No Residual samples were collected on Farm D. H1-H7 indicate the seven occasions on which Farm H was sampled; on sampling occasions H4-H7 only two Fresh and two Residual samples were collected.

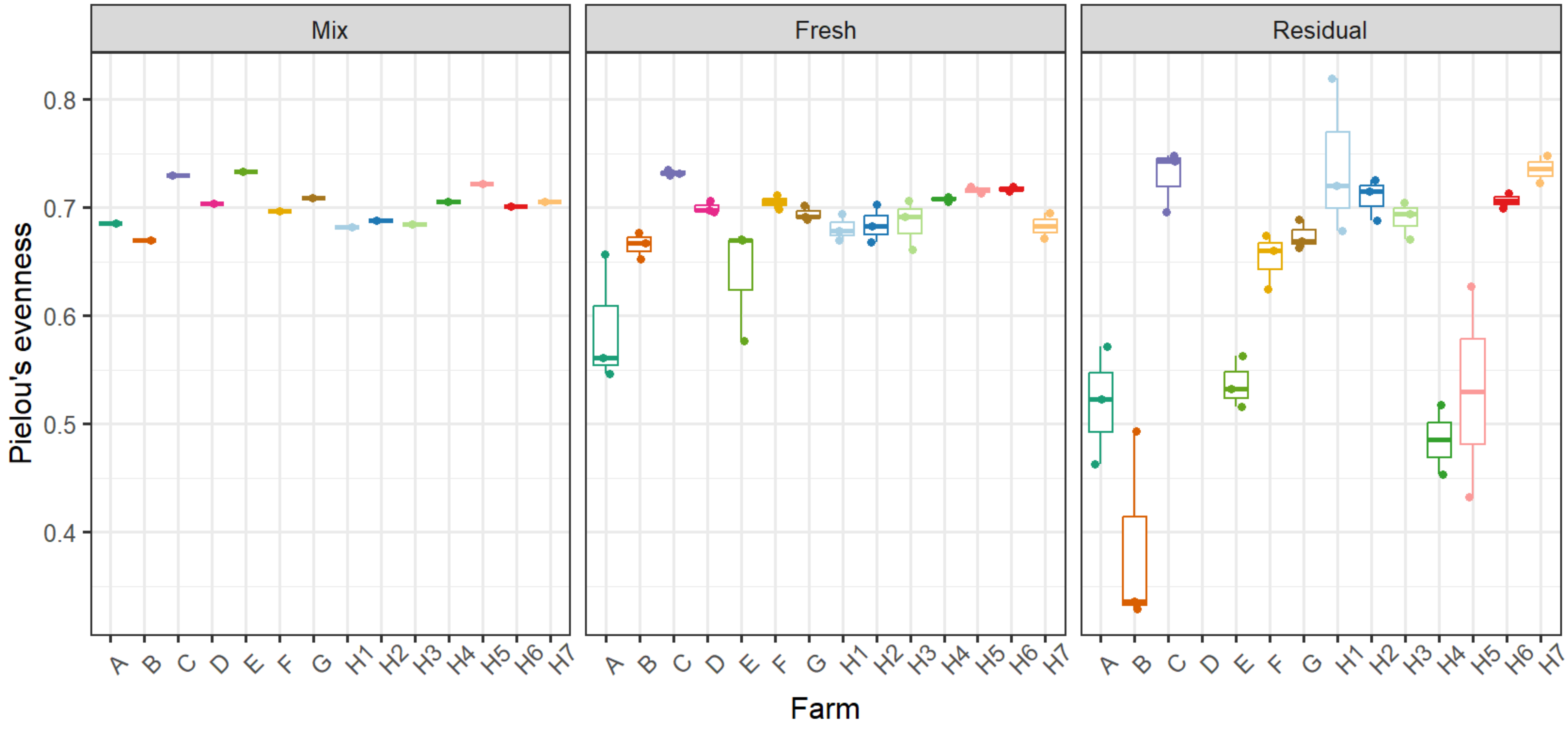

Supplementary Figure S5: Boxplots displaying Pielou's evenness of fungal taxa in liquid feed collected from each farm (A-H) at each respective sampling location; Mixing tank (Mix; *n* = 1), liquid feed sampled immediately after delivery to the troughs (Fresh; *n* = 3), liquid feed sampled prior to the next feed (Residual; *n* = 3). No Residual samples were collected on Farm D. H1-H7 indicate the seven occasions on which Farm H was sampled; on sampling occasions H4-H7 only two Fresh and two Residual samples were collected.

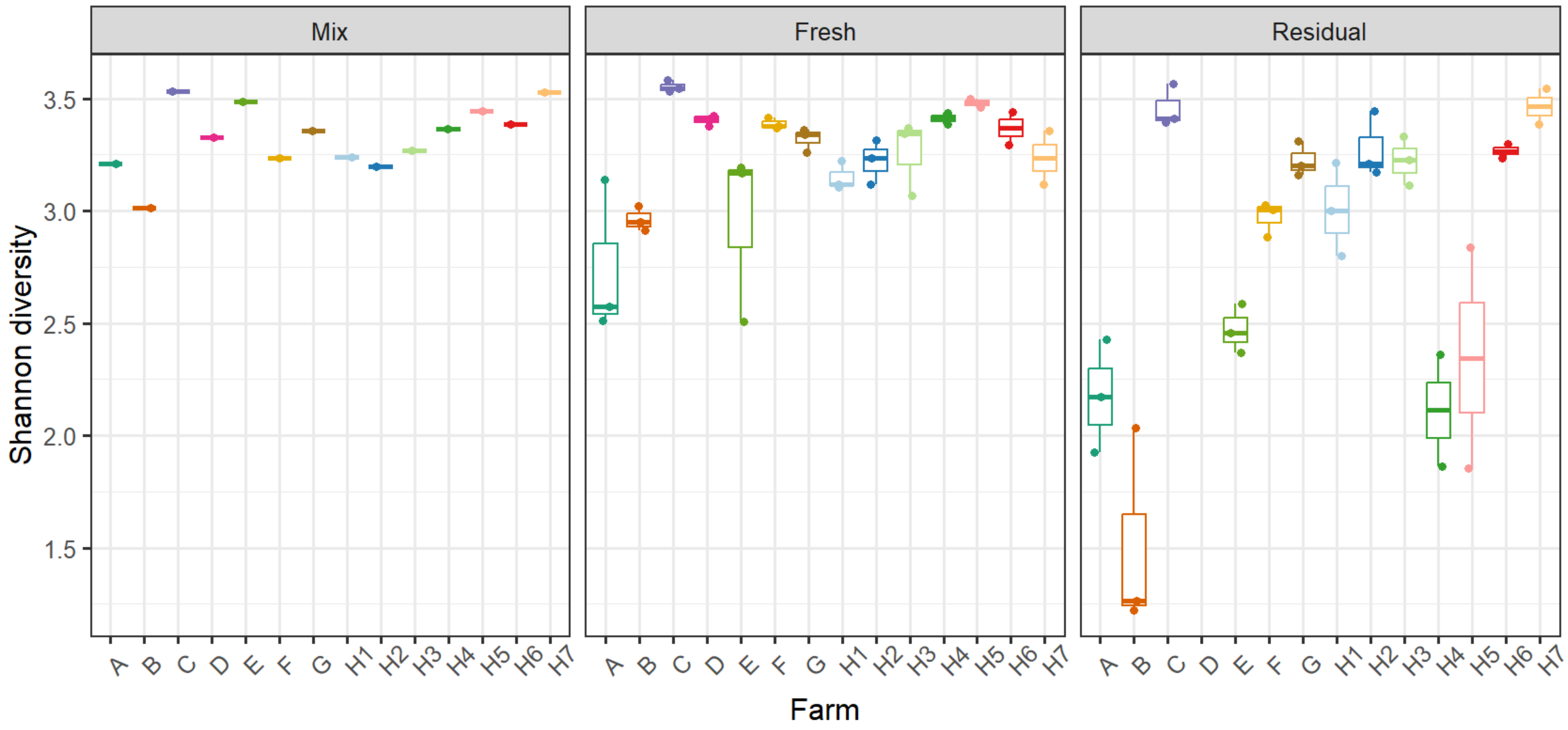

Supplementary Figure S6: Boxplots displaying Shannon diversity of fungal taxa in liquid feed collected from each farm (A-H) at each respective sampling location; Mixing tank (Mix; *n* = 1), liquid feed sampled immediately after delivery to the troughs (Fresh; *n* = 3), liquid feed sampled prior to the next feed (Residual; *n* = 3). No Residual samples were collected on Farm D. H1-H7 indicate the seven occasions on which Farm H was sampled; on sampling occasions H4-H7 only two Fresh and two Residual samples were collected.

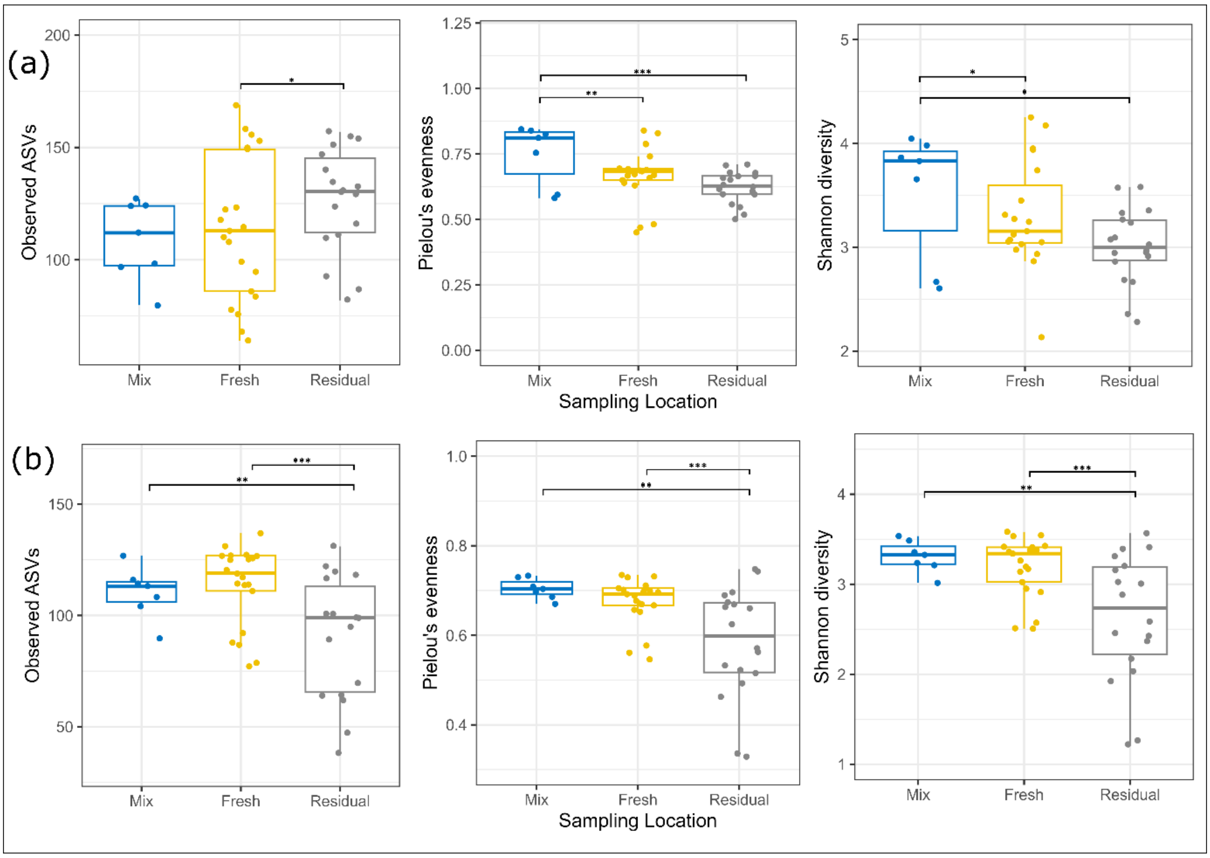

Supplementary Figure S7: Boxplots displaying observed amplicon sequence variants (ASVs), Pielou's evenness and Shannon diversity of bacterial (a) and fungal (b) communities in liquid feed samples from the mixing tank and in fresh and residual liquid feed samples from troughs on seven commercial pig production units; Mixing tank (Mix; $n = 7$), Fresh trough (Fresh; $n = 21$), Residual trough (Residual; $n = 18$). Data from Farm H has been omitted. No Residual samples were collected on Farm D. * $p \leq 0.05$, ** $p \leq 0.01$, *** $p \leq 0.001$.

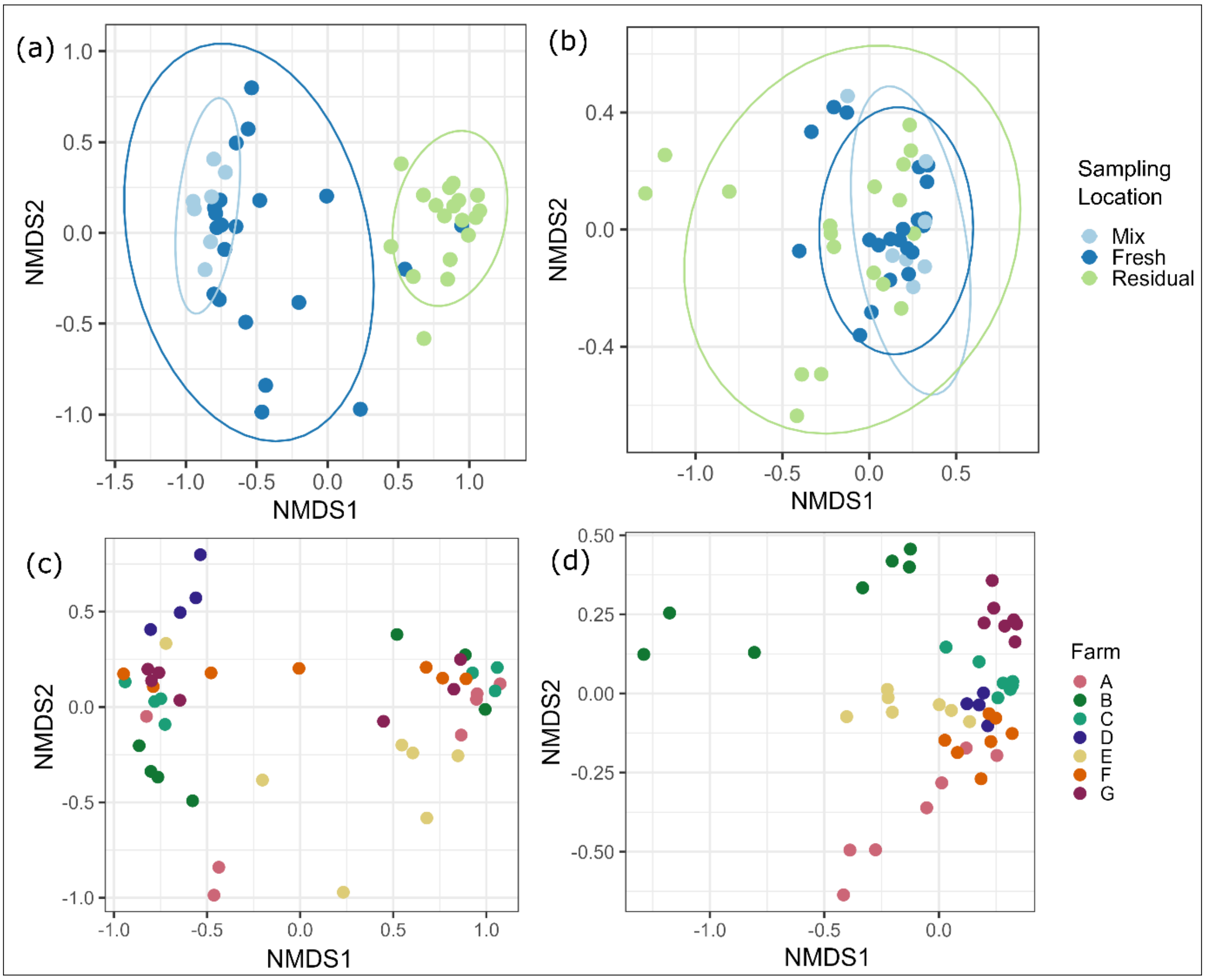

Supplementary Figure S8: Non-metric multidimensional scaling (NMDS) plots based on Bray-Curtis dissimilarity in liquid feed collected at each respective sampling location; Mixing tank (Mix; $n = 1$), liquid feed sampled immediately after delivery to the troughs (Fresh; $n = 3$), liquid feed sampled prior to the next feed (Residual; $n = 3$) on seven commercial pig units (data from Farm H has been omitted) for bacterial (a) and fungal (b) communities, and between farms for bacterial (c) and fungal (d) communities (excluding Farm H).

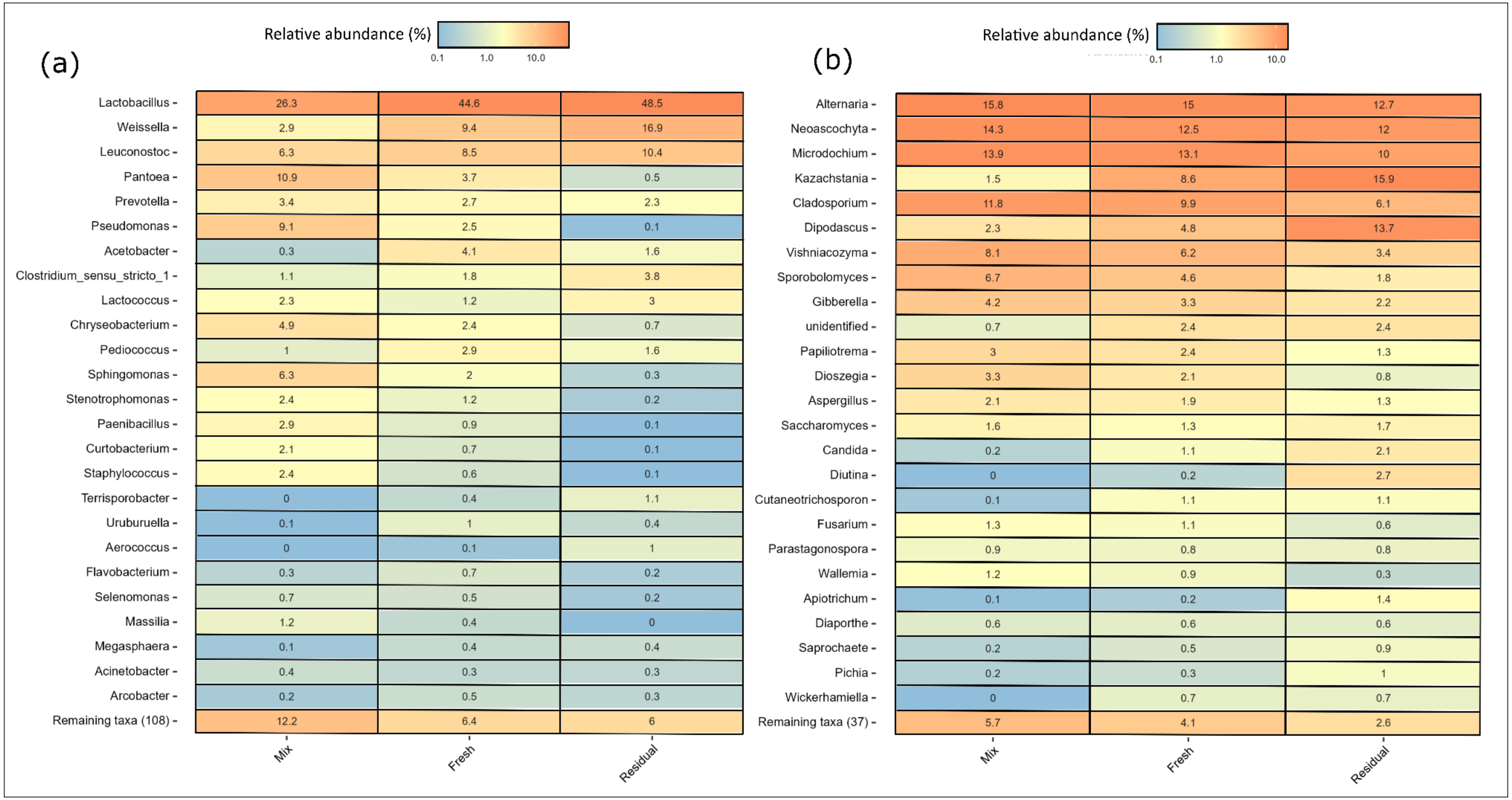

(a)	Mix	Fresh	Residual
Lactobacillus	26.3	44.6	48.5
Weissella	2.9	9.4	16.9
Leuconostoc	6.3	8.5	10.4
Pantoea	10.9	3.7	0.5
Prevotella	3.4	2.7	2.3
Pseudomonas	9.1	2.5	0.1
Acetobacter	0.3	4.1	1.6
Clostridium_sensu_stricto_1	1.1	1.8	3.8
Lactococcus	2.3	1.2	3
Chryseobacterium	4.9	2.4	0.7
Pediococcus	1	2.9	1.6
Sphingomonas	6.3	2	0.3
Stenotrophomonas	2.4	1.2	0.2
Paenibacillus	2.9	0.9	0.1
Curtobacterium	2.1	0.7	0.1
Staphylococcus	2.4	0.6	0.1
Terrisporobacter	0	0.4	1.1
Uruburuella	0.1	1	0.4
Aerococcus	0	0.1	1
Flavobacterium	0.3	0.7	0.2
Selenomonas	0.7	0.5	0.2
Massilia	1.2	0.4	0
Megasphaera	0.1	0.4	0.4
Acinetobacter	0.4	0.3	0.3
Arcobacter	0.2	0.5	0.3
Remaining taxa (108)	12.2	6.4	6

(b)	Mix	Fresh	Residual
Alternaria	15.8	15	12.7
Neoascochyta	14.3	12.5	12
Microdochium	13.9	13.1	10
Kazachstania	1.5	8.6	15.9
Cladosporium	11.8	9.9	6.1
Dipodascus	2.3	4.8	13.7
Vishniacozyma	8.1	6.2	3.4
Sporobolomyces	6.7	4.6	1.8
Gibberella	4.2	3.3	2.2
unidentified	0.7	2.4	2.4
Papiliotrema	3	2.4	1.3
Dioszegia	3.3	2.1	0.8
Aspergillus	2.1	1.9	1.3
Saccharomyces	1.6	1.3	1.7
Candida	0.2	1.1	2.1
Diutina	0	0.2	2.7
Cutaneotrichosporon	0.1	1.1	1.1
Fusarium	1.3	1.1	0.6
Parastagonospora	0.9	0.8	0.8
Wallemia	1.2	0.9	0.3
Apiotrichum	0.1	0.2	1.4
Diaporthe	0.6	0.6	0.6
Saprochaete	0.2	0.5	0.9
Pichia	0.2	0.3	1
Wickerhamiella	0	0.7	0.7
Remaining taxa (37)	5.7	4.1	2.6

Supplementary Figure S9: Heatmap displaying mean relative abundance (%) of the 25 most abundant bacterial (a) and fungal (b) genera in liquid feed between sampling locations. Data are the mean of values from all farms for each sampling location; mixing tank (Mix): *n* = 14; fresh feed from the trough (Fresh): *n* = 38, residual feed from the troughs (Residual): *n* = 35.
